# Endothelial BMP6 Drives Hemodynamic‐Dependent VSMCs Calcification in Carotid Atherosclerosis

**DOI:** 10.1002/advs.202502801

**Published:** 2025-10-13

**Authors:** Shen Li, Shuang Cao, Peipei Li, Feng Zhang, Gangfeng Ren, Xin Wang, Jiawei Zhao, Chen Liu, Yuan Gao, Jie Xu, Yongjun Wang, Zongping Xia, Yuming Xu

**Affiliations:** ^1^ The Department of Neurology the First Affiliated Hospital of Zhengzhou University Zhengzhou Henan 12636 China; ^2^ Tianjian Laboratory of Advanced Biomedical Sciences School of life sciences Zhengzhou University Zhengzhou Henan 12636 China; ^3^ NHC Key Laboratory of Prevention and treatment of Cerebrovascular Disease Henan Key Laboratory of Cerebrovascular Diseases Zhengzhou Henan 12636 China; ^4^ China National Clinical Research Center for Neurological Diseases Beijing 105738 China; ^5^ Department of Neurology Beijing Tiantan Hospital Capital Medical University Beijing 105738 China; ^6^ The Department of Integrated Traditional and Western Nephrology The First Affiliated Hospital of Zhengzhou University Zhengzhou Henan 12636 China; ^7^ The Clinical Systems Biology Laboratories of the First Affiliated Hospital of Zhengzhou University Zhengzhou Henan 12636 China

**Keywords:** carotid atherosclerosis, cell cross‐talk, hemodynamics, single‐cell RNA sequencing, vascular calcification

## Abstract

Carotid atherosclerosis (CAS) is a major contributor to ischemic stroke, with vascular calcification driving disease progression. However, the molecular mechanisms driving vascular calcification in CAS remain unelucidated. Previous studies have confirmed that bone morphogenetic proteins (BMPs) play essential roles in calcification; however, the regulatory mechanisms of BMP6 signaling in vascular calcification remain unclear. This study aims to investigate the role of BMP6 in vascular calcification in CAS and the underlying mechanisms. A subset of endothelial cells (ECs) with high BMP6 expression, which interacted with specific vascular smooth muscle cells (VSMCs) via the BMP signaling pathway, is identified using single‐cell RNA sequencing of human CAS plaques. In vitro experiments demonstrate BMP6‐induced osteogenic differentiation of VSMCs. Moreover, BMP6 activates the small mother against decapentaplegic (SMAD) signaling pathway by binding to the BMP6 receptor complex. Experimental results from endothelium‐specific BMP6 knockout (BMP6*^ECKO^* ApoE^−/−^) and overexpression mice confirm that BMP6 exacerbates vascular calcification, whereas its knockdown reduces calcific lesions. Additionally, disturbed flow conditions upregulate BMP6 expression by suppressing Krüppel‐like factor 4 and linking hemodynamic forces to BMP6‐mediated calcification. These findings suggest that BMP6 is a key regulator of vascular calcification in CAS, driven by EC–VSMC interactions and hemodynamic stress.

## Graphical abstract

The study demonstrates that endothelial cell (EC)‐derived BMP6 promotes the osteogenic differentiation of vascular smooth muscle cells (VSMCs) through cell‐cell interactions. Additionally, the researchers preliminarily explore the driving effect of hemodynamic factors on BMP6‐induced calcification and reveal the regulatory role of KLF4 on BMP6. The findings highlight BMP6 as a promising therapeutic target for mitigating vascular calcification in carotid artery stenosis (CAS).

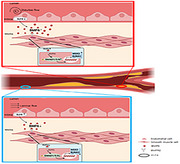

## 1 Introduction

Carotid atherosclerosis (CAS) is a significant cause of mortality and disability^[^ ^1^ ^]^ and accounts for up to 25% of ischemic strokes.^[^ ^2^, ^3^ ^]^ The primary pathological processes of CAS include carotid intima–media thickening, carotid plaque formation, vessel stenosis, and occlusion.^[^ ^1^, ^4^, ^5^ ^]^ Calcification contributes to luminal stenosis and the onset of ischemic symptoms. Several studies have demonstrated that calcification is present in ≈50–60% of carotid plaques.^[^ ^6^ ^]^ Although calcification is widely considered an indicator of CAS severity, its formation mechanisms and the molecular pathways regulating it remain poorly understood.

Vascular smooth muscle cells (VSMCs) play key roles in CAS‐related calcification.^[^ ^7^, ^8^, ^9^ ^]^ As the main component of the carotid media layer, VSMCs undergo transdifferentiation to form fibroblast‐like, macrophage‐like, osteogenic‐like, and adipocyte‐like subtypes in CAS.^[^ ^10^ ^]^

Increasing evidence suggests that osteogenic‐like transformation of VSMCs actively drives mineralization during vascular calcification.^[^ ^8^, ^11^ ^]^ Several special molecules have been shown to accelerate this phenotypic transformation, including matrix metalloproteinases,^[^ ^12^ ^]^ osteopontin,^[^ ^13^ ^]^ interleukin 6 (IL‐6),^[^ ^14^ ^]^ and bone morphogenetic proteins (BMPs).^[^ ^15^ ^]^ BMPs are members of the transforming growth factor (TGF)–β superfamily.^[^ ^16^ ^]^ Several studies have reported that BMP2, 4, and 7 are generally expressed in breast and prostate cancers.^[^ ^17^ ^]^ BMP9 protects against myocardial infarction by improving lymphatic drainage.^[^ ^18^ ^]^ Furthermore, BMP4 promotes vascular remodeling in hypoxic pulmonary hypertension.^[^ ^19^ ^]^ In the present study, a subtype of endothelial cells (ECs), which highly expresses BMP6, was identified using single‐cell techniques, and a BMP6 pathway network between ECs and VSMCs in CAS plaques was discovered. Studies on BMP6 have primarily focused on its roles in iron metabolism, cancer, and cardiovascular diseases.^[^ ^20^, ^21^, ^22^, ^23^ ^]^ Several studies have shown that EC‐derived BMP6 is involved in the regulation of iron metabolism by reducing the serum iron content.^[^ ^20^, ^21^ ^]^ In terms of tumor evolution, the BMP6–IL‐6 axis in mesenchymal stem cells drives the differentiation of acute myeloid leukemia cells.^[^ ^22^ ^]^ In addition, BMP6 has been proposed as a promising cardiac marker for predicting heart failure due to its involvement in cardiac fibrosis.^[^ ^23^ ^]^ However, the function of BMP6 in atherosclerosis remains unclear. Moreover, the stimuli that induce the high expression of BMP6 in ECs remain a key point of investigation.

Accumulating evidence indicates that disturbed flow and the resultant oscillatory shear stress (OSS) promote endothelial inflammation and oxidative damage, whereas laminar shear stress (LSS) maintains endothelial homeostasis by suppressing inflammatory pathways.^[^ ^24^, ^25^ ^]^ Krüppel‐like factor 4 (KLF4), a shear‐sensitive transcription factor predominantly expressed in ECs, is a key mediator of LSS‐dependent vascular protection.^[^ ^26^, ^27^ ^]^ Nevertheless, the potential link between KLF4‐mediated mechanotransduction and BMP6‐dependent calcification pathways requires further investigation.

This study aimed to explore the role of BMP6 in CAS, elucidate the regulatory mechanisms of BMP signaling in vascular calcification, and provide compelling evidence supporting BMP6 as a potential therapeutic target for CAS prevention.

## 2 Results

### 2.1 Single‐Cell RNA‐Sequencing (scRNA‐seq) of CAS Plaques

**Figure 1:**
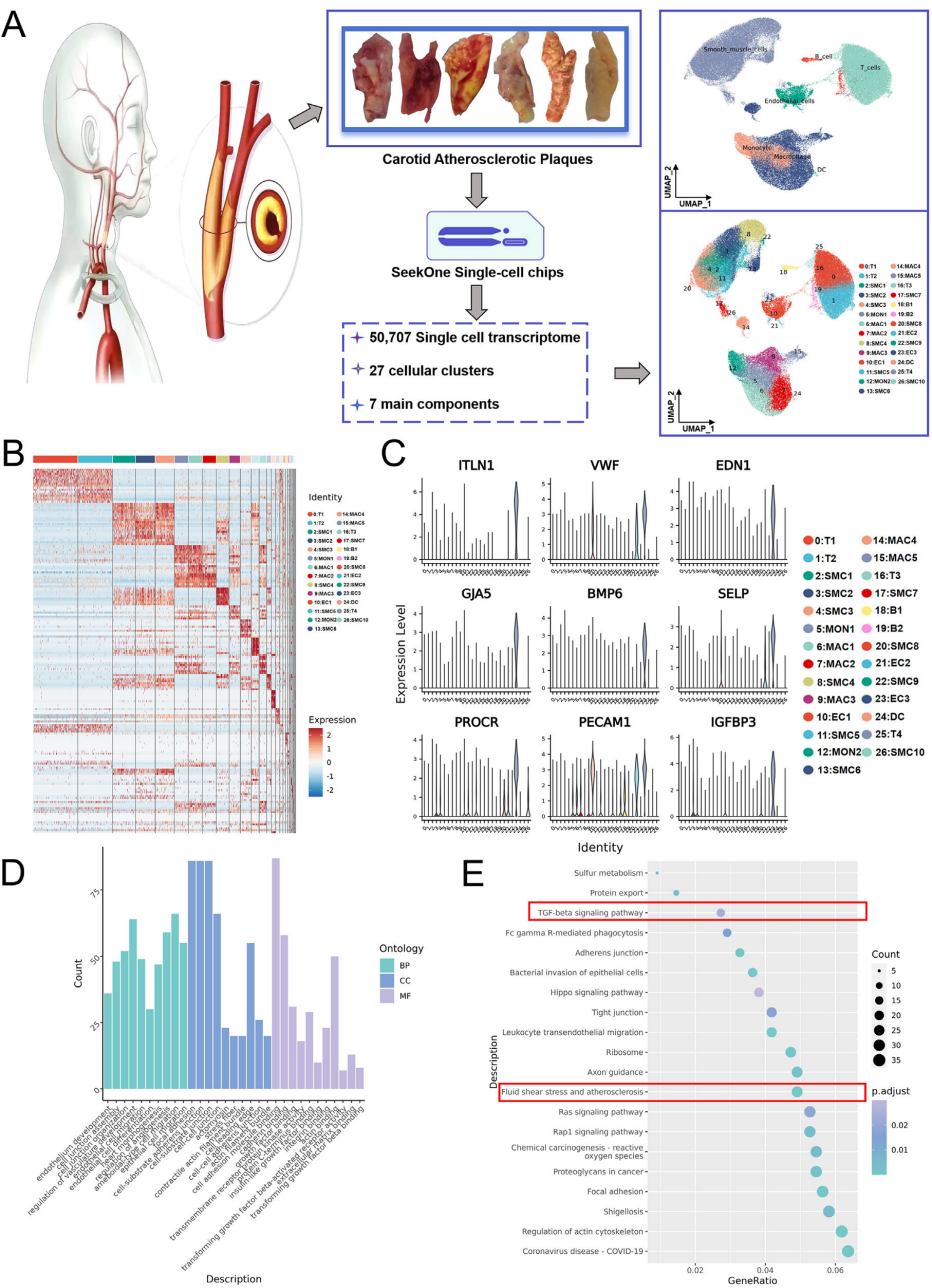
Single‐cell RNA‐sequencing (scRNA‐seq) of carotid atherosclerotic (CAS) plaques. A) Schematic illustration of the single‐cell study workflow for CAS plaques (*n* = 12). The left‐hand side shows a diagram of the human carotid artery. The middle part displays the partly obtained CAS plaque samples, which were analyzed using SeekOne single‐cell chips, resulting in 50707 single‐cell transcriptomes, 27 cellular clusters, and 7 main components. The right‐hand side presents different uniform manifold approximation and projection (UMAP) plots showing the distribution of cellular clusters. B) Heatmap presenting the gene expression profiles across different cellular clusters. Rows represent different genes, columns represent cellular clusters, and the color indicates gene expression levels. C) Violin plots showing the expression levels of specific genes in different cellular clusters. Each violin plot corresponds to a gene, and different colors represent different cellular clusters. D) Bar chart showing the cell count statistics for different ontology categories. The *X*‐axis represents the ontology descriptions, and the *Y*‐axis represents the count. E) Bubble plot displaying the results of gene enrichment analysis. The size and color of the bubbles reflect the gene count and *P*‐adjusted value, respectively, indicating the significance of enrichment for various biological processes (red frames indicate predominantly enriched pathways).

After applying quality control filters, 50707 cells from 12 CAS plaques obtained following carotid endarterectomy (CEA) were included in scRNA‐seq analysis. Gene expression data were aligned and projected in a 2D space using uniform manifold approximation and projections (UMAP) to identify distinct cell populations. To cluster cells based on gene expression levels, unsupervised Seurat‐based clustering was used, and 27 cell clusters were detected (**Figure** **1**A). Each cluster was identified using the top ten marker genes, as shown in Figure 1B. The CellMarker and PanglaoDB datasets were used to identify different cell types, such as ECs, VSMCs, T cells, and macrophages (Table S1, Supporting Information). The preliminary data suggested that BMP6 mutations could significantly increase the 3‐month and 1‐year risks of stroke recurrence in patients with large artery atherosclerosis^[^ ^28^ ^]^ (Figure S1, Supporting Information). Notably, BMP6 was highly expressed in the EC3 cluster (logFC = 3.25, *P* = 0; Figure 1C). However, the functional role of BMP6 in ECs remains unclear. Gene Ontology (GO) analysis of differentially overexpressed genes in EC3 suggested diverse functions such as endothelium development, regulation of vasculature development, and TGF‐β–activated receptor activity (Figure 1D). Kyoto Encyclopedia of Genes and Genomes (KEGG) functional enrichment analysis revealed that EC3 was associated with the TGF‐β signaling pathway and fluid shear stress and atherosclerosis (Figure 1E), suggesting a potential link between BMP6 and atherosclerosis.

**Figure 2:**
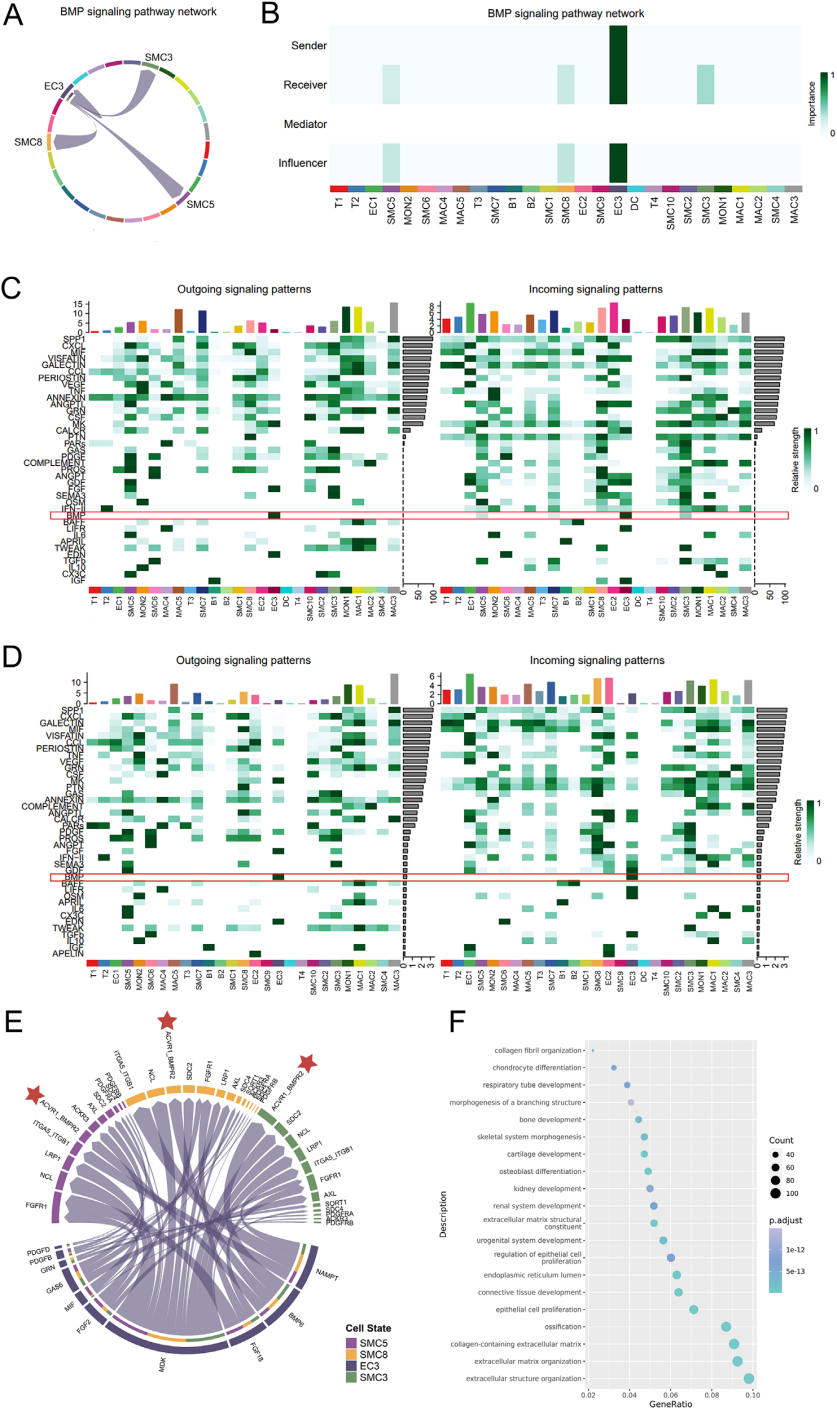
BMP signaling pathway network and enrichment analysis associated with the BMP signaling pathway. A) Chord diagram representing the BMP signaling pathway network, showing the relationships between different cell‐types. B) Grid‐based visualization of the BMP signaling pathway network, indicating the roles of the sender, receiver, mediator, and influencer among different cell‐types. The color represents the intensity of the signaling relationship. C, D) Communication network system displaying the outgoing and incoming signaling patterns of different genes in symptomatic (C) and asymptomatic patients with CAS (D). The left side of each communication network system shows the outgoing signaling patterns, whereas the right side shows the incoming signaling patterns. The color scale represents the magnitude of the signaling strength, and different cell‐types and genes are labeled at the top and left‐hand sides, respectively. Red frames indicate the signal strength of the BMP pathway in different cell clusters. E) Chord diagram showing BMP signaling pathways between EC3 and SMC3, SMC5, and SMC8 clusters. Asterisk indicates the primary common receptor in BMP signaling pathways. F) Bubble plot presenting the results of enrichment analysis related to the BMP signaling pathway. The size and color of the dots represent the count and *P‐*adjusted value, respectively, indicating the significance and the number of genes involved in each biological process. BMP, bone morphogenic protein; CAS, carotid atherosclerosis; EC, endothelial cell; SMC, smooth muscle cell.

To validate these findings, the cell–cell communication between EC3 and other cells was compared. Surprisingly, the EC3 cluster showed exclusive cell–cell communication with three specific smooth muscle cell (SMC) clusters—SMC3, SMC5, and SMC8—through the BMP signaling pathway, excluding interactions with itself (**Figure** **2**A). Moreover, the three SMC clusters exhibited distinct roles in the BMP signaling pathway network (Figure 2B). EC3, SMC5, and SMC8 functioned as both receivers and influencers of BMP6 signaling, regulating the transduction process of the signaling flow. However, these interactions depended on the stage of atherosclerosis. Communication network system analysis revealed enhanced BMP signaling in symptomatic patients with CAS, characterized by EC3 cells sending signals and EC3, SMC3, SMC5, and SMC8 cells receiving signals (Figure 2C). By contrast, no interactions between ECs and SMCs were observed within the plaques of the asymptomatic patients with CAS (Figure 2D). These findings suggested that BMP signaling promoted plaque progression. The chord diagram demonstrated that the BMP signaling pathways between EC3 cells and SMCs were primarily mediated by ligand binding between BMP6 and the ACVR1–BMPR2 receptor complex (Figure 2E). GO analysis of the differentially overexpressed genes in SMC5 suggested enrichment in ossification, osteoblast differentiation, and bone development (Figure 2F), suggesting that SMC5 may be a subtype of VSMCs transforming from the classical to the calcific subtype. These findings supported the hypothesis that BMP6 regulates the phenotypic transformation of SMCs.

### 2.2 Potential Function of BMP6 in CAS Plaques

**Figure 3:**
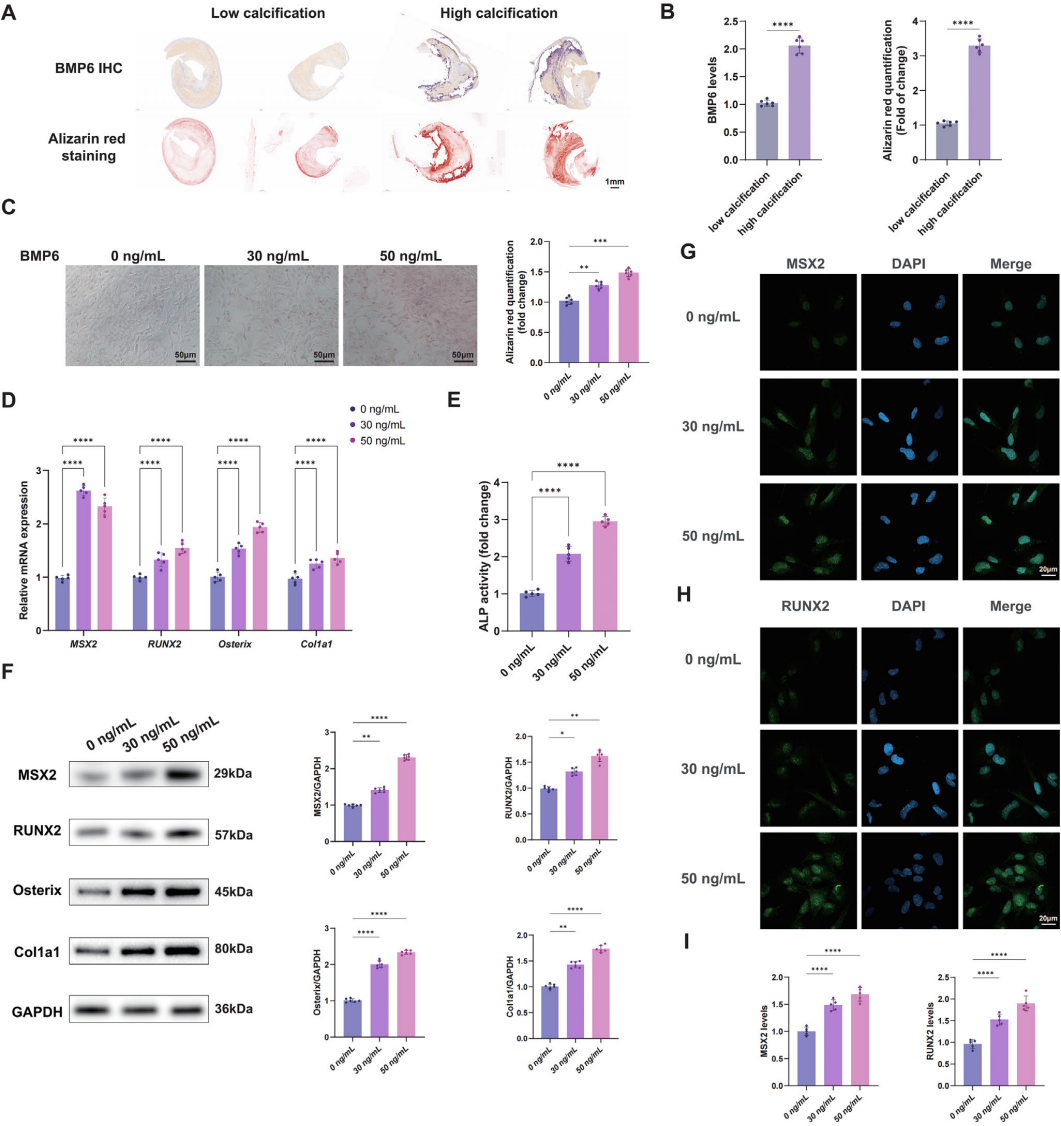
Potential function of BMP6 in CAS plaques. A) Representative image of low‐ and high‐calcification sections (*n* = 4); scale bar = 1mm. The sections show different degrees of BMP6 immunohistochemical staining (upper) and Alizarin Red staining (lower). B) Quantitative analysis of BMP6 levels and Alizarin Red staining in low‐ and high‐calcification groups (*n* = 6). C) Microscopic images of VSMCs cultured in the osteogenic medium for 3 weeks and treated with different BMP6 concentrations (0, 30, and 50 ng mL^−1^) for 4 days; scale bar = 50µm. Quantitative analysis of Alizarin Red staining (*n* = 6). D) Relative mRNA expression levels of MSX2, RUNX2, Osterix, and Col1a1 in VSMCs treated with different concentrations of BMP6 (*n* = 5). E) Detection of ALP activity in VSMCs treated with different concentrations of BMP6 (*n* = 5). F) Western blot analysis of MSX2, RUNX2, Osterix, and Col1a1 protein levels in VSMCs treated with different concentrations of BMP6 (*n* = 6). GAPDH was used as a loading control. G–I) Immunofluorescence staining images of MSX2 (G) and RUNX2 (H) in VSMCs treated with different concentrations of BMP6; scale bar = 20µm. Quantification data (I) of RUNX2 and MSX2 immunofluorescence intensity (*n* = 5). Data are presented as mean ± standard deviation, and *P*‐values were determined using the unpaired two‐tailed Student's *t*‐tests (B) or one‐way ANOVAs followed by Tukey's test (C‐F, I); ^*^ *p* < 0.05, ^**^ *p* < 0.01, ^***^ *p* < 0.001, and ^****^ *p* < 0.0001. IHC, immunohistochemistry; BMP, bone morphogenic protein; MSX2, Msh Homeobox 2; RUNX2, runt‐related transcription factor 2; Col1a1, collagen type I alpha 1; ALP, alkaline phosphatase; GAPDH, glyceraldehyde‐3‐phosphate dehydrogenase; DAPI, 4′,6‐diamidino‐2‐phenylindole.

Previous studies have predominantly used Alizarin Red staining to assess the degree of calcification.^[^ ^29^, ^30^ ^]^ The plaque samples collected after CEA revealed that both immunohistochemical staining for BMP6 and Alizarin Red staining were significantly more intense in high‐calcification plaques than in low‐calcification plaques, highlighting a strong association between BMP6 expression and the degree of calcification (**Figure** **3**A). Quantitative analysis further confirmed that BMP6 expression was significantly elevated in highly calcified plaques (Figure 3B). To further explore this correlation in vitro, VSMCs were cultured in an osteogenic medium and treated with varying concentrations of BMP6. Alizarin Red staining revealed that the transdifferentiation of contractile‐like VSMCs into osteogenic‐like VSMCs was enhanced in a dose‐dependent manner (Figure 3C), suggesting that BMP6 actively promotes osteogenesis. Moreover, the significant upregulation of collagen type I alpha 1 and common osteogenic markers, such as Msh Homeobox 2 (MSX2), runt‐related transcription factor 2 (RUNX2), and Osterix, was observed, as determined by quantitative reverse transcription polymerase chain reaction (RT‐qPCR) and Western blot analyses (Figure 3D,F). Furthermore, treatment of VSMCs with BMP6 significantly enhanced alkaline phosphatase (ALP) activity (Figure 3E). Immunofluorescence staining further confirmed the enhanced nuclear localization of MSX2 and RUNX2 in response to BMP6 stimulation (Figure 3G–I). Collectively, these findings demonstrate that BMP6 plays a central role in promoting calcification in CAS and facilitates the osteogenic transdifferentiation of VSMCs.

### 2.3 EC‐Derived BMP6 Promotes the Osteogenic Differentiation of VSMCs

**Figure 4:**
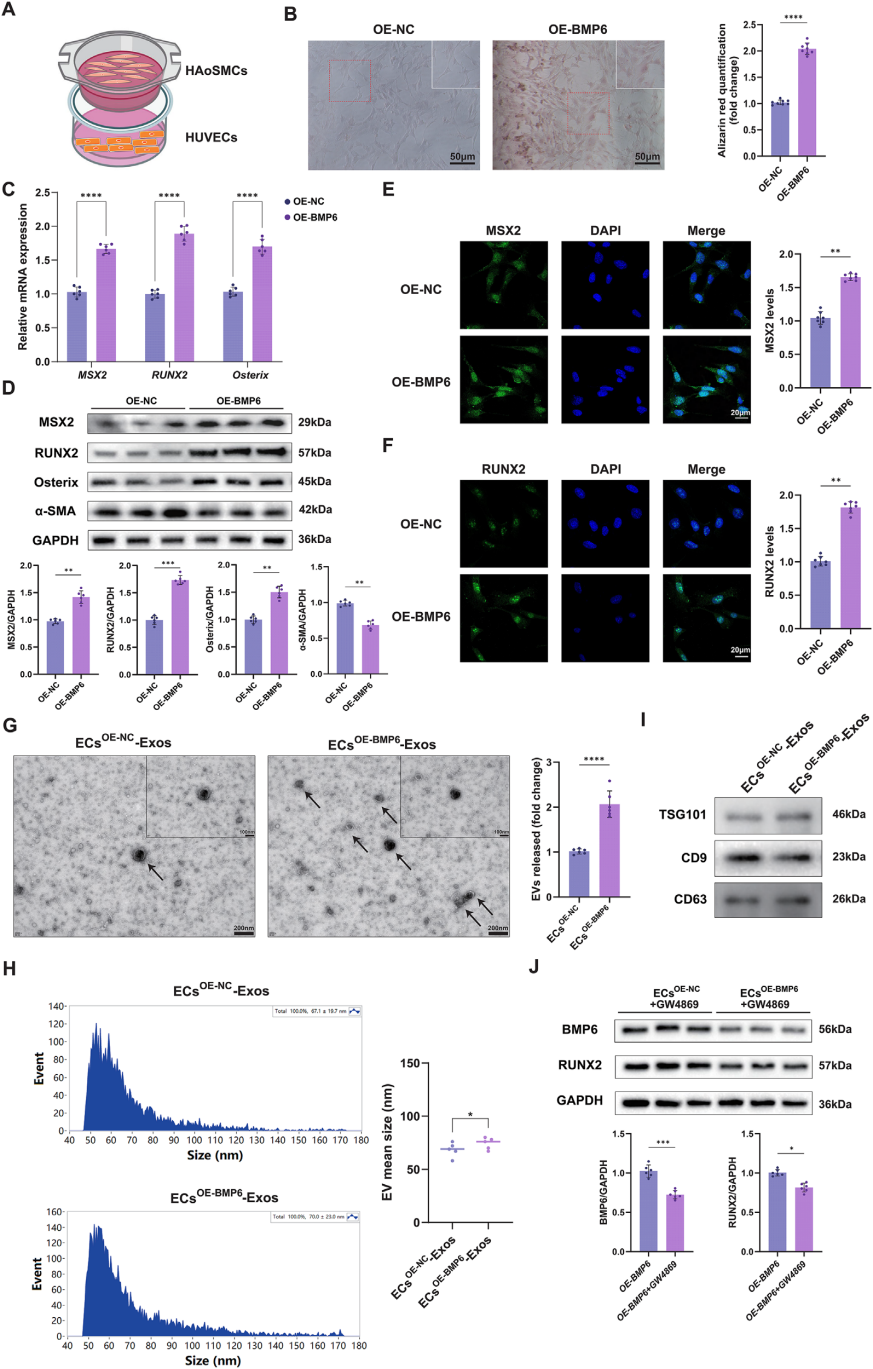
EC‐derived BMP6 promotes the osteogenic differentiation of VSMCs. A) Schematic illustration of the co‐culture system of HAoSMCs and HUVECs. HUVECs in the lower chamber, transfected 24 h earlier with BMP6 over‐expression (OE‐BMP6) plasmid or empty vector control (OE‐NC) plasmid, were co‐cultured with HAoSMCs in the upper chamber for 48 h using six‐well transwell units. B) HAoSMCs in the upper chamber are harvested to conduct Alizarin red staining. Representative images of Alizarin red staining of HAoSMCs in the OE‐NC group and OE‐BMP6 groups. The inset in the upper right corner shows a magnified view of the boxed area; scale bar = 50µm. Quantification of Alizarin red staining is presented as a fold change compared with the OE‐NC group (*n* = 8). C) HAoSMCs in the upper chamber are harvested to determine the relative mRNA expression levels of MSX2, RUNX2, and Osterix in the OE‐NC and OE‐BMP6 groups (*n* = 6). D) HAoSMCs in the upper chamber are harvested to determine the protein expression levels of MSX2, RUNX2, Osterix, and α‐SMA in the OE‐NC and OE‐BMP6 groups. GAPDH was used as a loading control. Quantification of these protein levels is shown (*n* = 6). E,F) HAoSMCs in the upper chamber are harvested to conduct immunofluorescence staining of MSX2 (E) and RUNX2 (F) in the OE‐NC and OE‐BMP6 groups; scale bar = 20 µm. Quantification data of MSX2 and RUNX2 immunofluorescence intensity are shown (*n* = 7). G) EVs from the co‐cultured medium were isolated using ultracentrifugation. Representative images of EVs isolated from ECs^OE‐NC^‐Exos and ECs^OE‐BMP6^‐Exos are visualized using transmission electron microscopy (the target is indicated by the arrows); scale bar = 200nm (lower) and 100nm (upper). Exosomes release is quantified, shown on the right side (*n* = 6). H) Nanoparticle tracking analysis of exosome size distribution for ECs^OE‐NC^‐Exos (upper) and ECs^OE‐BMP6^‐Exos (lower), and the average particle size (right) of two groups (*n* = 5). I) Western blot of exosomal markers TSG101, CD9, and CD63 in ECs^OE‐NC^‐Exos and ECs^OE‐BMP6^‐Exos. J) Western blot analysis of BMP6 and RUNX2 expression in HAoSMCs co‐cultured with GW4869‐pretreated ECs^OE‐NC^ or ECs^OE‐BMP6^. GAPDH was used as a loading control. Quantitative analysis of relative protein expressions is shown below (*n* = 6). Data are presented as means ± standard deviation, and *P*‐values were determined using the unpaired two‐tailed Student's *t*‐tests; ^*^ *p* < 0.05, ^**^ *p* < 0.01, ^***^ *p* < 0.001, and ^****^ *p* < 0.0001. BMP, bone morphogenic protein; HAoSMCs, human aortic smooth muscle cells; HUVECs, human umbilical vein endothelial cells; EV, Extracellular vesicle; MSX2, Msh Homeobox 2 (MSX2); RUNX2, runt‐related transcription factor 2; α‐SMA, α‐smooth muscle actin; TSG101, tumor susceptibility gene 101; GAPDH, glyceraldehyde‐3‐phosphate dehydrogenase; DAPI, 4′,6‐diamidino‐2‐phenylindole; VSMCs, vascular smooth muscle cells.

Through the preliminary analysis of single‐cell transcriptomic sequencing data for plaque samples, an EC cluster with high BMP6 expression and its interaction with specific SMC clusters were identified, which might promote the phenotypic transformation of VSMCs. To further explore this in vitro, BMP6 was overexpressed in human umbilical vein endothelial cells (HUVECs) (Figure S2A,B, Supporting Information), which were then co‐cultured with human aortic SMCs (HAoSMCs) in a transwell chamber (**Figure** **4**A). Alizarin red staining confirmed that HUVEC‐derived BMP6 augmented calcium deposition in co‐cultured HAoSMCs (Figure 4B). The expressions of MSX2, RUNX2, and Osterix were significantly elevated, but α‐smooth muscle actin (α‐SMA) was reduced in co‐cultured HAoSMCs (Figure 4C,D). Immunofluorescence staining revealed a similar trend, corroborating the role of BMP6 in promoting osteogenic activity (Figure 4E,F). These results demonstrated that EC‐derived BMP6 could potentially promote the osteogenic differentiation of VSMCs.

Additionally, extracellular vesicles (EVs) from the co‐culture medium of HUVECs and HAoSMCs were isolated via ultracentrifugation. Transmission electron microscopy revealed that EVs were spherical with a characteristic double‐layer membrane structure. Interestingly, the number of ECs^OE‐BMP6^‐Exos vesicles was nearly twofold higher than that of ECs^OE‐NC^‐Exos vesicles (Figure 4G). The exosome protein yield and particle number per milliliter were higher in ECs^OE‐BMP6^‐Exo than in ECs^OE‐NC^‐Exo (Figure S3A,B, Supporting Information). The two groups showed no significant difference in the number of particles per 100 µg of protein (Figure S3C, Supporting Information). Nanoparticle tracking analysis revealed that the mean diameters of vesicles in ECs^NC^‐Exo and ECs^OE‐BMP6^‐Exo were 67.1 ± 19.7 and 70.0 ± 23.0, respectively, which exhibited a typical single sharp peak (Figure 4H). Western blot analysis further showed that the vesicles were positive for the surface marker proteins CD9 and CD63, as well as the tumor susceptibility gene 101 (Figure 4I). However, when HUVECs were incubated with GW4869 prior to co‐culture with HAoSMCs, the expression of BMP6 and RUNX2 was significantly blocked (Figure 4J). These results led us to hypothesize that EC‐BMP6 drives SMCs ossification, possibly in the form of exosomes.

### 2.4 BMP6 Activates Phosphorylated SMAD 1/5/8 Signaling During the Osteogenic Differentiation of VSMCs

**Figure 5:**
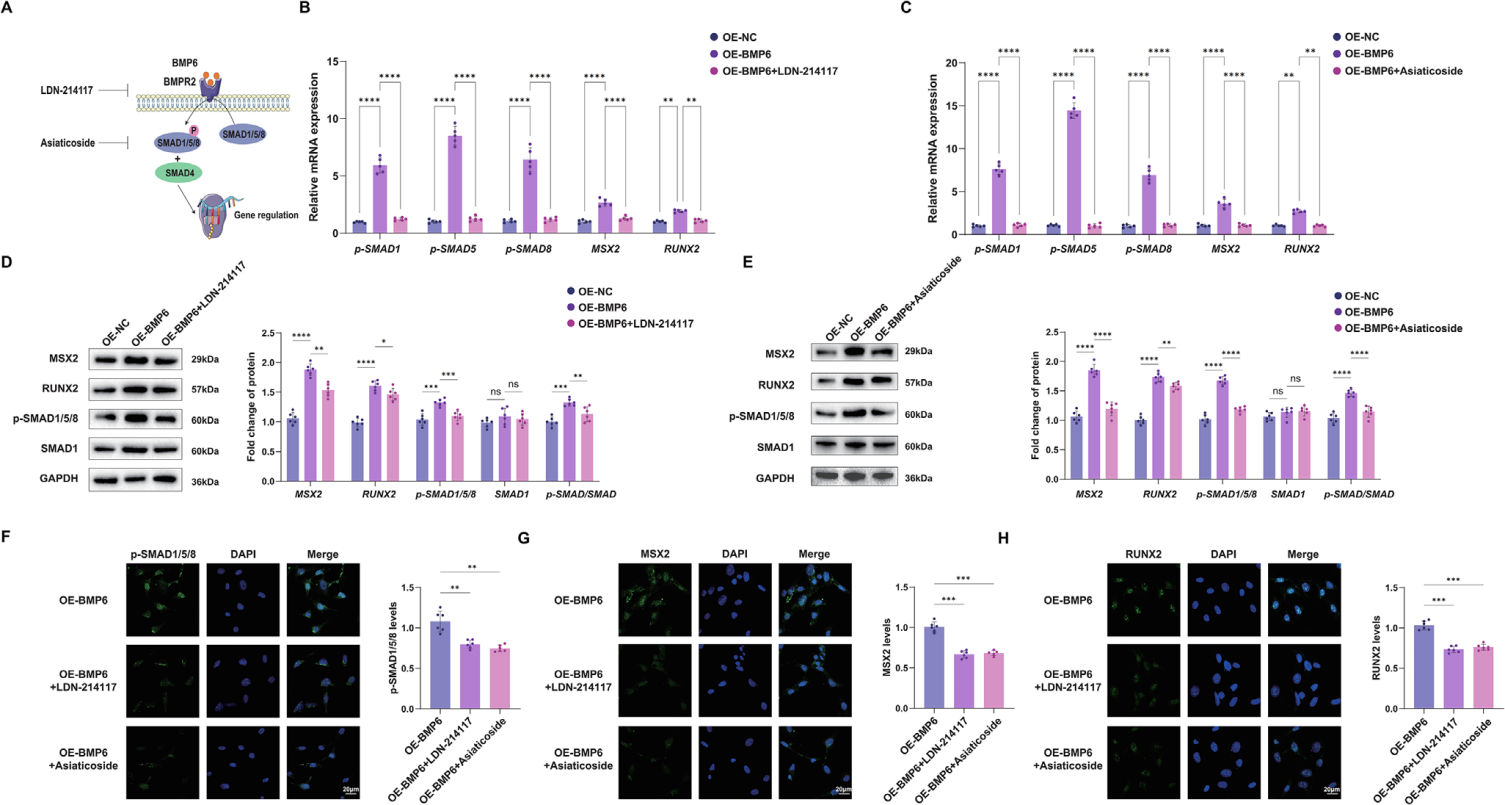
BMP6 activates the phosphorylated SMAD 1/5/8 signaling pathway during the osteogenic differentiation of VSMCs. A) Schematic diagram showing the signaling pathway involving BMP6, BMPR2, and the subsequent activation of SMADs and gene regulation, with LDN‐214117 and asiaticoside acting as inhibitors. B) Relative mRNA expression levels of p‐SMAD1, p‐SMAD5, p‐SMAD8, MSX2, and RUNX2 in the control, OE‐BMP6, and OE‐BMP6+LDN‐214117 groups (*n* = 5). C) Western blot analysis of MSX2, RUNX2, p‐SMAD1/5/8, and SMAD1 protein levels in the control, OE‐BMP6, and OE‐BMP6+LDN‐214117 groups. GAPDH was used as a loading control. Quantification of these protein levels is shown (*n* = 6). D) Relative mRNA expression levels of p‐SMAD1, p‐SMAD5, p‐SMAD8, MSX2, and RUNX2 in the control, OE‐BMP6, and OE‐BMP6+asiaticoside groups (*n* = 5). E) Western blot analysis of MSX2, RUNX2, p‐SMAD1/5/8, and SMAD1 protein levels in the control, OE‐BMP6, and OE‐BMP6+asiaticoside groups. GAPDH was used as a loading control. Quantification of these protein levels is shown (*n* = 6). F–H) Immunofluorescence staining of p‐SMAD1/5/8 (F), MSX2 (G), and RUNX2 (H) in the OE‐BMP6, OE‐BMP6+LDN‐214117, and OE‐BMP6+asiaticoside groups; scale bar = 20 µm. Quantification of the immunofluorescence intensity is presented below each panel (*n* = 6). Data are presented as mean ± standard deviation, and *P*‐values were determined using the one‐way ANOVAs followed by Tukey's test; ns, no significant difference, ^*^ *p* < 0.05, ^**^ *p* < 0.01, ^***^ *p* < 0.001, and ^****^ *p* < 0.0001. BMP, bone morphogenic protein; MSX2, Msh Homeobox 2; RUNX2, runt‐related transcription factor 2; GAPDH, glyceraldehyde‐3‐phosphate dehydrogenase; DAPI, 4′,6‐diamidino‐2‐phenylindole; VSMCs, vascular smooth muscle cells; OE, over‐expression.

Bioinformatics analysis demonstrated an interaction between EC3 and SMC5 mediated by BMP6 and the ACVR1–BMPR2 receptor complex, contributing to its pro‐osteogenic activity (Figure 2). To validate this finding, co‐cultured HAoSMCs were categorized into the control, BMP6 (OE‐BMP6), and OE‐BMP6+LDN‐214117 groups. LDN‐214117, a BMPR2 inhibitor, was used to reduce the binding of BMP6 to the ACVR1–BMPR2 receptor complex in vitro. The results showed decreased expression of the osteogenic markers, RUNX2 and MSX2, after LDN‐214117 treatment (**Figure** **5**A–C). SMAD proteins are essential for nuclear transduction of BMPs. Surprisingly, the transcription and expression of p‐SMAD1/5/8 decreased after LDN‐214117 treatment. These results suggested that the pro‐osteogenic effect of BMP6 could be mediated through its binding to the ACVR1–BMPR2 receptor complex, subsequently activating the p‐SMAD signaling pathway. Asiaticoside, a p‐SMAD signaling inhibitor, was used to silence p‐SMAD1/5/8 signaling. HAoSMCs following co‐culture were categorized into the control, OE‐BMP6, and OE‐BMP6+asiaticoside groups. Asiaticoside was observed to potentially attenuate the expression of p‐SMAD and other key osteogenic markers (Figure 5A,D,E). Similarly, treatment with LDN‐214117 and asiaticoside significantly reduced the expression of these markers, as indicated by fluorescence intensity measurements (Figure 5F–H). These results supported the hypothesis that BMP6 promoted osteogenesis by binding to BMPR2 and activating the p‐SMAD1/5/8 signaling pathway.

### 2.5 ECs‐Derived BMP6 Increases Vascular Calcification in Apolipoprotein E‐Deficient (ApoE^−/−^) Mice with Western Diet

**Figure 6:**
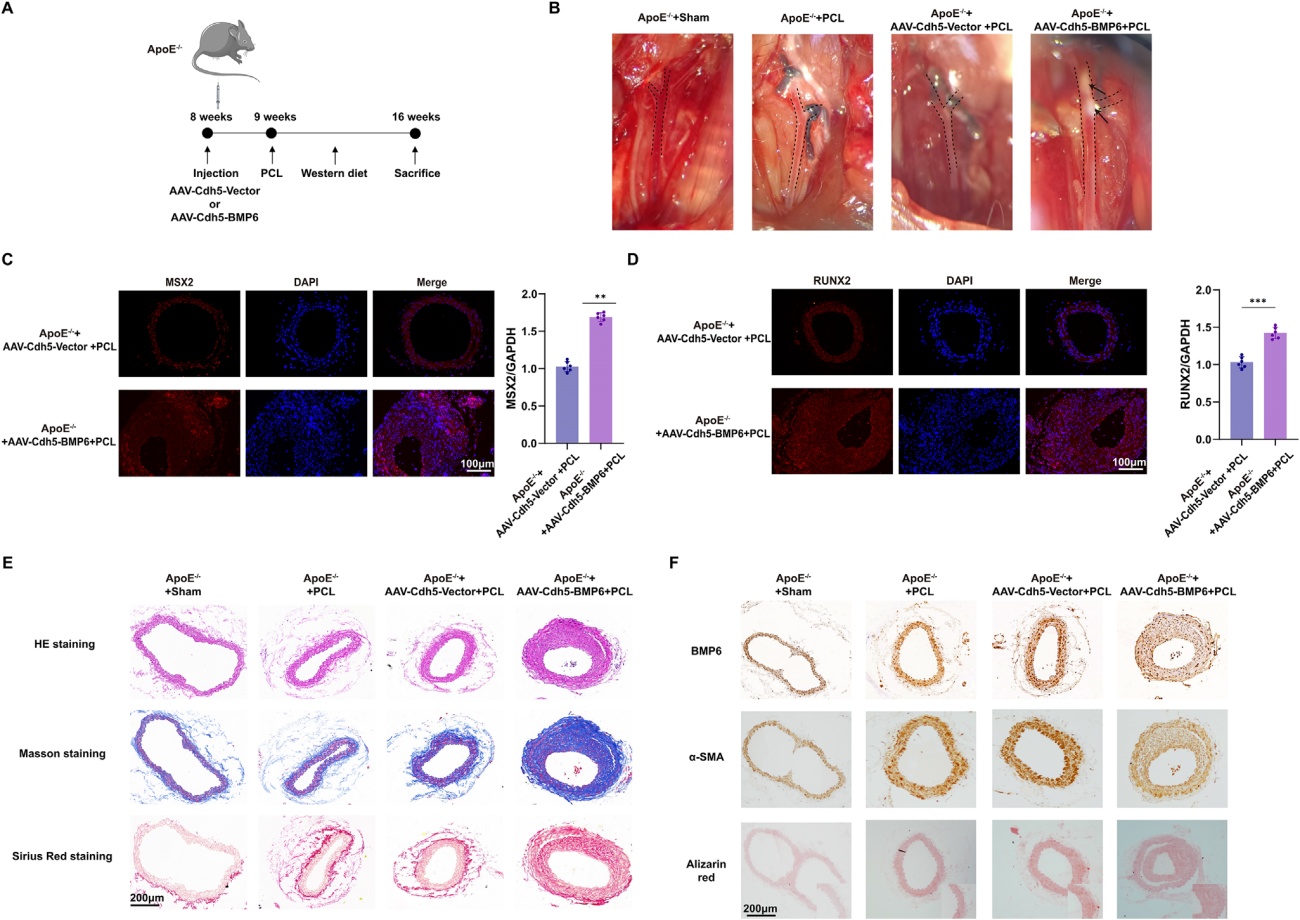
EC‐derived BMP6 increases vascular calcification in ApoE^−/−^ mice with Western diet. A) Diagram showing ApoE^−/−^ mice being injected with AAV‐Cdh5‐Vector or AAV‐Cdh5‐BMP6 via the tail vein on the eighth week, subjected to PCL within 1 week, and fed a Western diet until sixteenth week. B) Representative images of blood vessels in the ApoE^−/−^+sham, ApoE^−/−^+PCL, ApoE^−/−^+AAV‐Cdh5‐Vector+PCL, and ApoE^−/−^+AAV‐Cdh5‐BMP6+PCL groups (the dashed outline indicates the carotid artery; the arrow points to the atherosclerotic plaque). C,D) Immunofluorescence staining to detect the expression of MSX2 (C) and RUNX2 (D) in the ApoE^−/−^+AAV‐Cdh5‐Vector+PCL and ApoE^−/−^+AAV‐Cdh5‐BMP6+PCL groups; scale bar = 100 µm. Quantitative analysis of fluorescence intensity is presented (*n* = 6). E) Histological staining images of the carotid arteries in ApoE^−/−^+sham, ApoE^−/−^+PCL, ApoE^−/−^+AAV‐Cdh5‐Vector+PCL, and ApoE^−/−^+AAV‐Cdh5‐BMP6+PCL mice, including hematoxylin–eosin, Masson, and Sirius Red staining (from left to right); scale bar = 200 µm. F) Immunohistochemical staining of the carotid arteries in ApoE^−/−^+sham, ApoE^−/−^+PCL, ApoE^−/−^+AAV‐Cdh5‐Vector+PCL, and ApoE^−/−^+AAV‐Cdh5‐BMP6+PCL mice, showing BMP6, α‐SMA, and Alizarin Red staining (from left to right); scale bar = 200 µm. Data are presented as means ± standard deviation, and *P‐*values were determined using the unpaired two‐tailed Student's *t*‐tests; ^**^ *p* < 0.01 and ^***^ *p* < 0.001. ApoE, apolipoprotein E; EC, endothelial cell; PCL, partial carotid artery ligation; BMP, bone morphogenic protein; α‐SMA, α‐smooth muscle actin; DAPI, 4′,6‐diamidino‐2‐phenylindole; MSX2, Msh Homeobox 2; RUNX2, runt‐related transcription factor 2.

To corroborate the aforementioned in vivo findings, an animal model of EC‐specific BMP6 overexpression was established (Figure S4, Supporting Information). Eight‐week‐old ApoE^−/−^mice were injected with AAV‐Cdh5‐BMP6 or AAV‐Cdh5‐Vector, followed by partial carotid ligation (PCL) and Western diet to induce vascular calcification (**Figure** **6**A; Figure S5, Supporting Information). Gross morphological observations revealed more severe calcification in ApoE^−/−^+AAV‐Cdh5‐BMP6 mice than in ApoE^−/−^+AAV‐Cdh5‐Vector mice (Figure 6B). Upregulated expression of osteogenic markers, including RUNX2 and MSX2, was also detected in ApoE^−/−^ mice with BMP6 overexpression. Quantitative analysis confirmed elevated levels of MSX2 and RUNX2 in ApoE^−/−^+AAV‐Cdh5‐BMP6 mice (*p* < 0.01 for MSX2, and *p* < 0.001 for RUNX2; Figure 6C,D). Hematoxylin–eosin, Masson, and Sirius Red staining revealed thickened vascular walls, increased extracellular matrix deposition, and enhanced collagen accumulation in ApoE^−/−^+AAV‐Cdh5‐BMP6 mice (Figure 6E). To verify the role of BMP6 in promoting the phenotypic transformation of VSMCs, the expression levels of BMP6 and contractile SMC marker α‐SMA were compared. Immunohistochemical staining indicated higher BMP6 expression and lower α‐SMA expression in the vascular walls of ApoE^−/−^+AAV‐Cdh5‐BMP6 mice. Furthermore, Alizarin Red staining confirmed a marked increase in calcium deposition in the vascular tissues of ApoE^−/−^ mice injected with AAV‐Cdh5‐BMP6 (Figure 6F). Collectively, these findings suggested that endothelial‐specific overexpression of BMP6 expedited vascular calcification by promoting calcium accumulation and osteogenic differentiation of VSMCs.

### 2.6 Endothelial‐Specific BMP6 Knockdown Decreases Vascular Calcification in ApoE^−/−^ Mice with Western Diet

**Figure 7:**
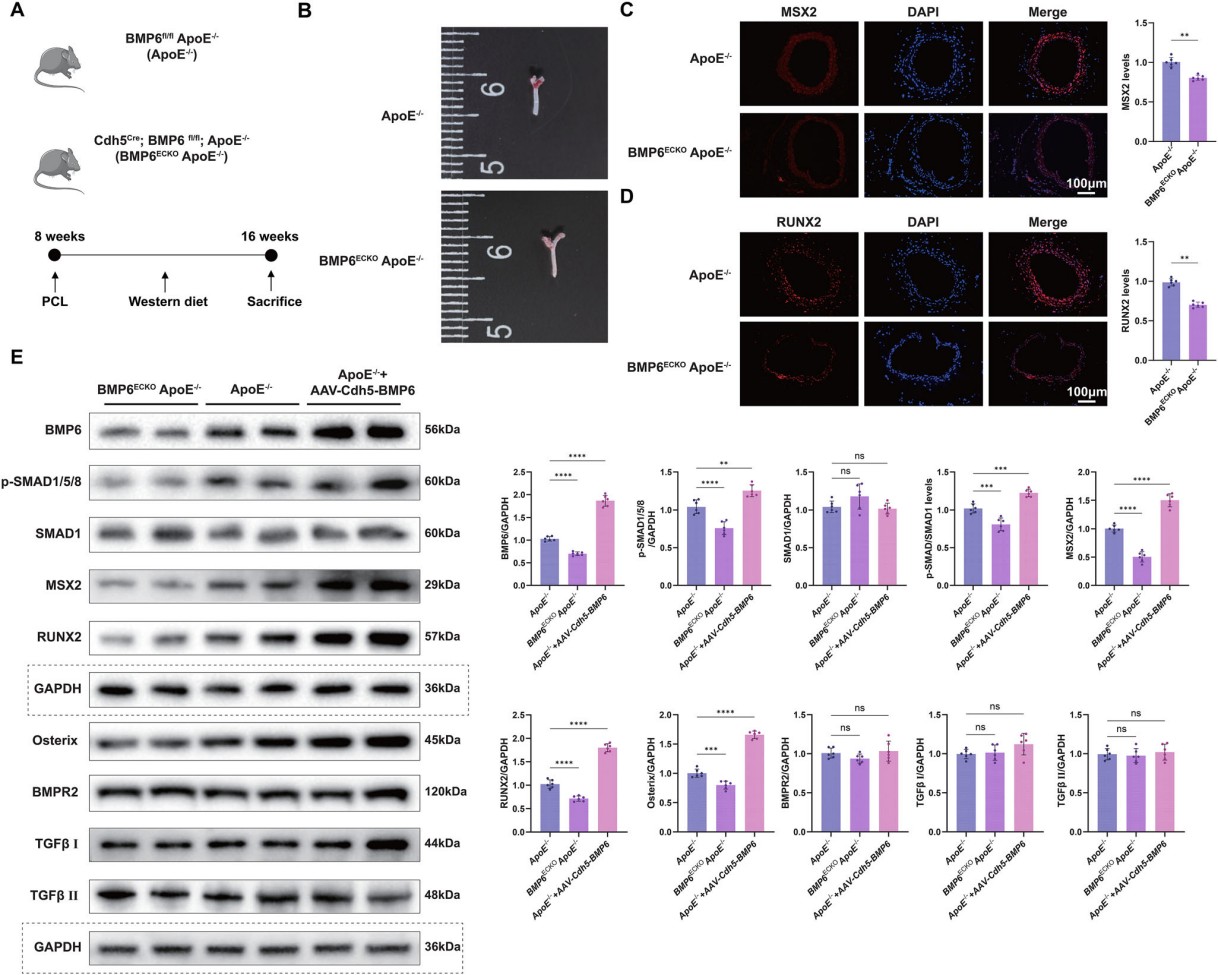
Endothelial‐specific BMP6 knockdown decreased vascular calcification in ApoE^−/−^ mice with a Western diet. A) Schematic illustration of the experimental procedures for two mouse models—ApoE^−/−^ and *Cdh5^Cre^; BMP6^fl/fl^*; ApoE^−/−^ (BMP6*^ECKO^* ApoE^−/−^). PCL was administered on the eighth week, and a Western diet was provided for 8 weeks, following which the mice were sacrificed. B) Oil Red O staining of the carotid artery in ApoE^−/−^ and BMP6*^ECKO^* ApoE^−/−^ mice. C,D) Immunofluorescence staining for the detection of MSX2 (C) and RUNX2 (D) expression in ApoE^−/−^ and BMP6*^ECKO^* ApoE^−/−^ mice; scale bar = 100 µm. Quantitative analysis results of fluorescence intensity are presented (*n* = 6). E) Western blot analysis of the protein expression levels of BMP6, p‐SMAD1/5/8, SMAD1, MSX2, RUNX2, Osterix, BMPR2, TGFβ I, and TGFβ II in ApoE^−/−^, BMP6*^ECKO^* ApoE^−/−^, and ApoE^−/−^+AAV‐Cdh5‐BMP6 mice. GAPDH was used as a loading control (Dashed box). Quantitative analysis data of the corresponding protein levels are shown on the right side (*n* = 6). Data are presented as mean ± standard deviation, and *P*‐values were determined using the unpaired two‐tailed Student's *t*‐tests (C,D) or one‐way ANOVAs followed by Tukey's test (E); ns, no significant difference, ^**^ *p* < 0.01, ^***^ *p* < 0.001, and ^****^ *p* < 0.0001. ApoE, apolipoprotein E; BMP, bone morphogenic protein; PCL, partial carotid ligation; DAPI, 4′,6‐diamidino‐2‐phenylindole; MSX2, Msh Homeobox 2; RUNX2, runt‐related transcription factor 2; BMPR2, bone morphogenic protein receptor 2; TGFβ I, transforming growth factor β I; TGFβ II, transforming growth factor β II; GAPDH, glyceraldehyde‐3‐phosphate dehydrogenase.

To understand the potential of BMP6 as a therapeutic target in vascular calcification, endothelial‐specific BMP6 in ApoE^−/−^ mice was knocked out using the CRISPR/Cas9 genome editing approach (BMP6*^ECKO^* ApoE^−/−^) (Figure S6A,B, Supporting Information). These mice, along with BMP6*^fl/fl^* ApoE^−/‐^ (ApoE^−/−^) mice, were subjected to PCL and Western diet regimens (**Figure** **7**A). The carotid arteries were harvested from BMP6*^ECKO^* ApoE^−/‐^ and ApoE^−/‐^ mice and subjected to Oil Red O staining to visualize atherosclerotic plaques. The results showed that vascular atherosclerosis was noticeably more diminished in BMP6*^ECKO^* ApoE^−/−^ mice than in ApoE^−/−^ mice (Figure 7B). Immunofluorescence staining and quantitative analysis indicated that MSX2 and RUNX2 were significantly downregulated in the carotid arteries of BMP6*^ECKO^* ApoE^−/−^ mice (*p* < 0.0001, Figure 7C,D). Furthermore, relatively decreased levels of MSX2, RUNX2, Osterix, and p‐SMAD1/5/8 were observed in BMP6*^ECKO^* ApoE^−/−^ mice and elevated expression in ApoE^−/−^+AAV‐Cdh5‐BMP6 mice compared to those in control ApoE^−/−^ mice. By contrast, the levels of BMPR2 and TGF‐β I and II showed no significant differences in these mice (Figure 7E). Furthermore, GW4869, a pharmacological compound known to effectively inhibit exosome biogenesis and release, was used to validate the hypothesis that BMP6 was transferred via exosomes in ApoE^−/−^+AAV‐Cdh5‐BMP6 mice. Western blotting and quantitative analyses revealed reduced expression of RUNX2, MSX2, and Osterix in the GW4869 pretreatment group (Figure S7A,B, Supporting Information). These results demonstrate the function of endothelial BMP6 in osteogenic differentiation and the activation of the p‐SMAD1/5/8 signaling pathway.

### 2.7 Disturbed Flow Induces BMP6 Expression by the Up‐Regulated KL4 Transcription Factor

**Figure 8:**
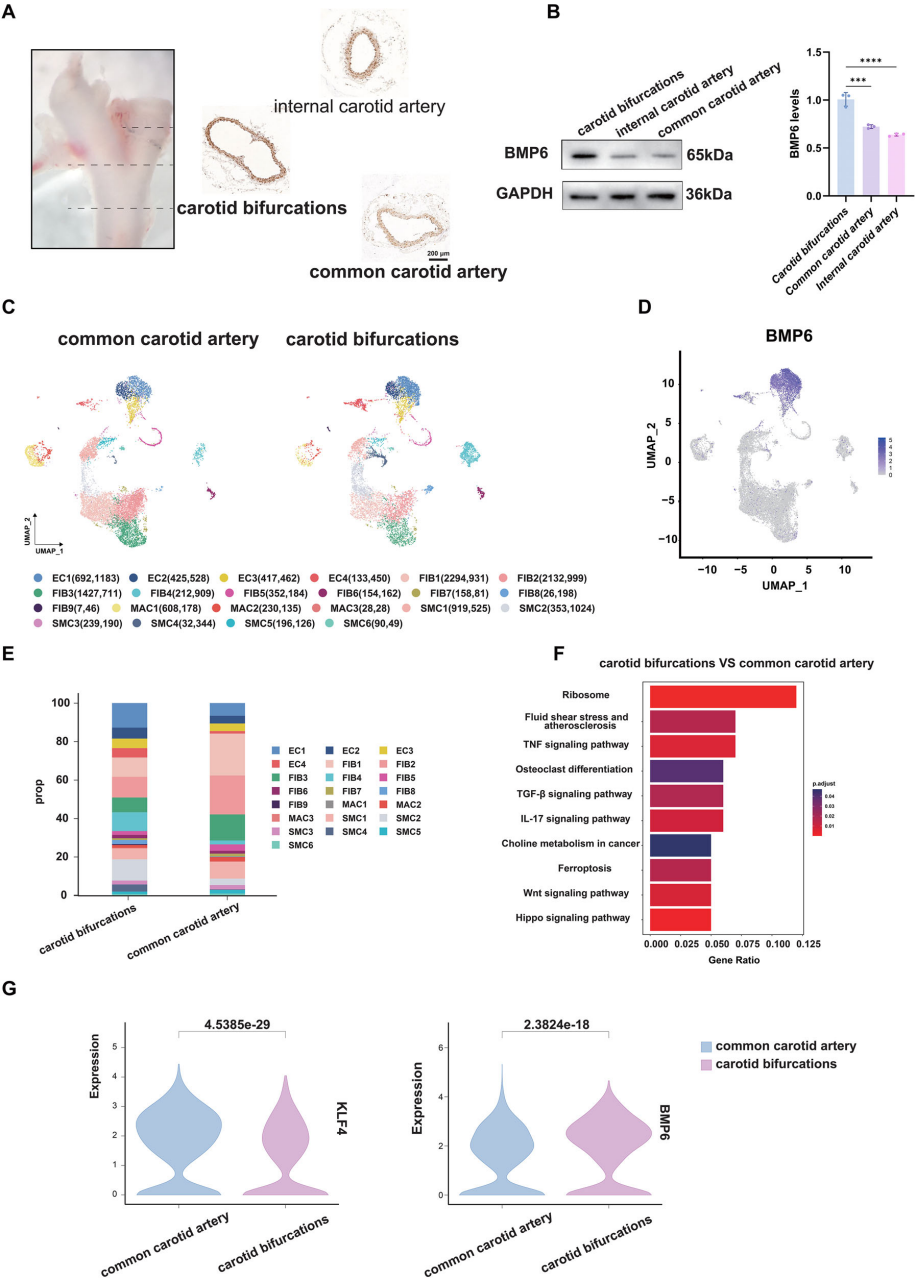
Distribution of BMP6 in the common carotid artery and carotid bifurcations. A) Images showing the anatomical location of carotid bifurcations, the common carotid artery, and the internal carotid artery in mice, along with immunohistochemical staining images of BMP6 in these regions; scale bar = 200 µm. B) Western blot analysis of BMP6 protein expression in carotid bifurcations, the common carotid artery, and the internal carotid artery. GAPDH was used as a loading control. Quantification data of BMP6 expression are presented (*n* = 3). C) UMAP plots showing the results of the cell distribution between the common carotid artery and carotid bifurcations. Different cell types are color‐coded. D) UMAP plots showing the distribution of BMP6. E) Stacked bar plot depicting the variation in cell proportions between the two groups. F) Bar graph displaying the KEGG pathway enrichment for genes upregulated at carotid bifurcations compared with those in the common carotid artery in the EC1 cluster. G) Violin plots depicting the expression levels of KLF4 and BMP6 in ECs from the common carotid artery and carotid bifurcations. Data are presented as mean ± standard deviation, and *P*‐values were determined using the one‐way ANOVAs followed by Tukey's test; ^***^ *p* < 0.001 and ^****^ *p* < 0.0001. BMP, bone morphogenic protein; GAPDH, glyceraldehyde‐3‐phosphate dehydrogenase; KLF4, Krüppel‐like factor 4; UMAP, uniform manifold approximation and projection.

**Figure 9:**
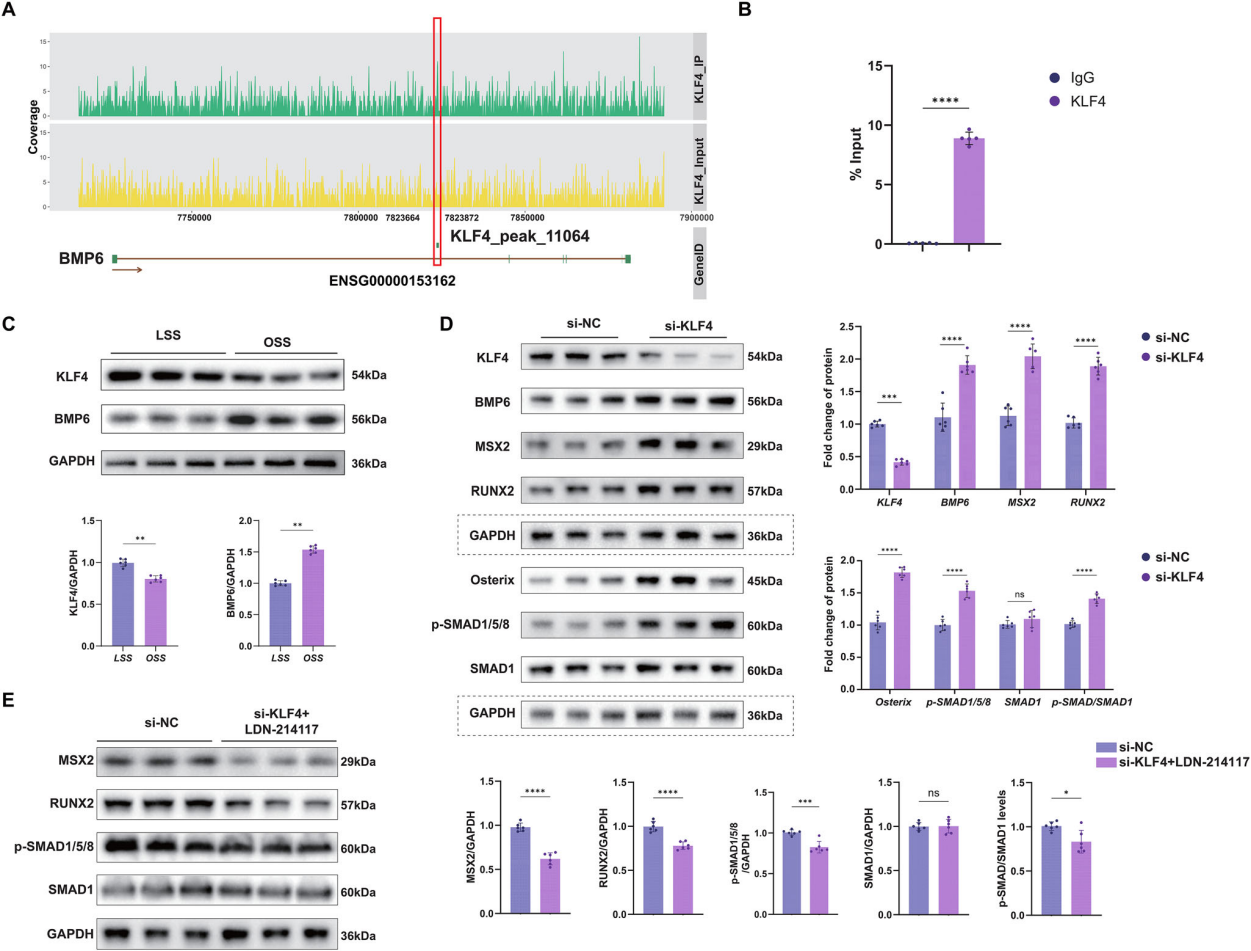
Disturbed flow initiates BMP6 expression by the up‐regulated KL4 transcription factor. A) KLF4 binding profile at the BMP6 locus. KLF4 ChIP‐seq track (red) illustrates enrichment at the BMP6 gene region, with Input as the normalization control. B) ChIP‐qPCR validation of KLF4 binding to the BMP6 promoter. Enrichment of KLF4 at the BMP6 promoter was quantified using ChIP‐qPCR, with IgG used as the negative control (*n* = 5). C) Western blot analysis of KLF4 and BMP6 protein expression under low LSS and OSS conditions. GAPDH was used as a loading control. Quantification data of protein expression levels are shown (*n* = 6). D) Western blot analysis of the protein expression levels of KLF4, BMP6, MSX2, RUNX2, Osterix, p‐SMAD1/5/8, and SMAD1 in HAoSMCs co‐cultured with HUVECs transfected with si‐NC (negative control small interfering RNA [siRNA]) and si‐KLF4. GAPDH was used as a loading control (dashed box). Quantification data of protein expression levels are presented on the right (*n* = 6). E) Western blot analysis of MSX2, RUNX2, p‐SMAD1/5/8, and SMAD1 in HAoSMCs co‐cultured with HUVECs transfected with si‐KLF4 and treated with or without LDN‐214117. GAPDH was used as a loading control. Quantification data of protein expression levels are presented (*n* = 6). Data are presented as mean ± standard deviation, and *P*‐values were determined using the unpaired two‐tailed Student's *t*‐tests; ns, no significant difference, ^*^ *p* < 0.05, ^**^ *p* < 0.01, ^***^ *p* < 0.001, and ^****^ *p* < 0.0001. KLF4, Krüppel‐like factor 4; BMP, bone morphogenic protein; MSX2, Msh Homeobox 2; RUNX2, runt‐related transcription factor 2; GAPDH, glyceraldehyde‐3‐phosphate dehydrogenase; LSS, laminar shear stress; OSS, oscillatory shear stress.

The bifurcation region of the carotid artery is highly prone to plaque calcification,^[^ ^31^ ^]^ which is a common site of OSS. Notably, BMP6 was observed to be specifically highly expressed in the carotid artery bifurcation than in the internal and common carotid arteries (**Figure** **8**A,B). These results suggested that BMP6 expression was possibly modulated by hemodynamics. To investigate the underlying molecular mechanism, carotid arteries from wild‐type mice were collected and divided into different regions for scRNA‐seq analysis. After batch effect removal, quality control, dimensionality reduction, clustering, and annotation of the data (Figure 8C; Figure S8A,B, Supporting Information), ECs were observed to be the predominant cell type expressing BMP6, especially EC1 (Figure 8D; Figure S8C, Supporting Information). The expression of BMP6 significantly differed between the common carotid artery and carotid bifurcations in the EC1 cluster (Figure 8E). Furthermore, fluid shear stress and atherosclerosis pathways were enriched in differentially expressed genes between the two regions according to KEGG analyses (Figure 8F). This finding also indicated that the expression of BMP6 might be related to hemodynamics. Therefore, the role of KLF4, a master regulator of shear stress‐responsive genes in ECs, was examined in relation to the site‐specific effects of blood flow on calcification.^[^ ^26^, ^27^ ^]^ The violin plots display that the expression of KLF4 and BMP6 exhibited opposite trends (Figure 8G). To understand the potential relationship between KLF4 and BMP6, chromatin immunoprecipitation (ChIP) sequencing was performed to explore whether KLF4 mediated the expression of BMP6. KLF4 was observed to potentially directly bind to the promoter of BMP6 (**Figure** **9**A,B; Figure S9A–C, Supporting Information). To further verify this correlation, a hydrodynamic device was used to simulate the effects of different flow patterns on vascular calcification in vitro (Figure S10A–D, Supporting Information). Using the cell fluid culture system, OSS stimulation was observed to significantly decrease KLF4 expression and increase BMP6 expression in HUVECs, whereas LSS stimulation showed the opposite results (Figure 9C). Using small interfering RNA, the expression of KLF4 was reduced in HUVECs; however, the effect of EC‐derived BMP6 on promoting vascular calcification was enhanced (Figure 9D). Moreover, the same manifestations were detected in endothelial‐specific KLF4 knockout mice (Figure S11A,B, Supporting Information). LDN‐214117 treatment reduced p‐SMAD1/5/8, MSX2, and RUNX2 levels in HAoSMCs co‐cultured with KLF4‐knockdown HUVECs, indicating that KLF4 modulates BMP‐SMAD signaling via a BMPR2‐dependent pathway (Figure 9E). These results illustrated that disturbed flow conditions, particularly OSS, enhanced BMP6 expression in ECs through a KLF4‐dependent mechanism.

## 3 Discussion

To our knowledge, this is the first study to comprehensively elucidate the pivotal role of BMP6 in vascular calcification associated with CAS. The findings revealed that EC‐derived BMP6 promoted the osteogenic differentiation of VSMCs via cell–cell interactions and explored the driving effect of hemodynamic factors on the calcification effect of BMP6, revealing the regulatory role of KLF4 on BMP6. The study findings highlighted BMP6 as a promising therapeutic target for mitigating vascular calcification in CAS.

Vascular calcification is a complex, multifactorial process characterized by the pathological deposition of calcium phosphate crystals within the arterial wall, leading to reduced vascular compliance and an increased risk of adverse events.^[^ ^13^, ^32^ ^]^ Several members of the BMP family have been implicated in the pathogenesis of vascular diseases. For instance, BMP9 regulates the osteoblastic differentiation of VSMCs in chronic kidney disease,^[^ ^33^ ^]^ BMP2 mediates pro‐osteogenic reprogramming in aortic valve interstitial cells,^[^ ^34^ ^]^ and BMP4 promotes the recruitment and activation of monocytes and macrophages, enhances foam cell formation, and affects inflammation and plaque stability.^[^ ^35^ ^]^ Previous studies have indicated that BMP6 plays multifaceted roles in iron metabolism, tumor biology, and cardiovascular disease, all of which may collectively contribute to vascular dysfunction. In iron metabolism, BMP6‐mediated hepcidin regulation affects iron homeostasis, and iron overload promotes ferroptosis and oxidative stress, contributing to endothelial dysfunction.^[^ ^20^, ^21^ ^]^ In tumors, BMP6 has been shown to modulate angiogenesis through VEGFR2 signaling and endothelial‐mesenchymal transition.^[^ ^36^ ^]^ Extending upon these findings, the present study provides the first evidence of the role of BMP6 in the pathogenesis of atherosclerosis, particularly in vascular calcification. The study findings demonstrate that EC‐derived BMP6 promotes the osteogenic differentiation of VSMCs by binding to the heteromeric ACVR1–BMPR2 receptor complex, which triggers the phosphorylation and nuclear translocation of SMAD1/5/8 transcription factors, ultimately upregulating the expression of osteogenic marker genes, such as RUNX2, MSX2, and Osterix. Moreover, animal experimental results demonstrated that vascular calcification was significantly worsened by endothelial‐specific overexpression of BMP6 but was alleviated by conditional knockout in ECs. These findings suggest that BMP6 is a critical mediator in vascular calcification pathogenesis.

Hemodynamic changes play a crucial role in both physiological and pathological processes of blood vessels, and different flow patterns can influence the function and gene expression of ECs.^[^ ^24^, ^25^ ^]^ Notably, this study linked flow dynamics to calcification by demonstrating that OSS upregulated BMP6 expression in ECs by suppressing KLF4 transcription. Previous studies have indicated that KLF4 is a flow‐sensitive transcription factor primarily expressed in ECs of the vascular wall.^[^ ^27^, ^37^ ^]^ The study findings revealed that disturbed blood flow suppresses KLF4 expression, which is consistent with the results of previous studies. In vitro flow‐chamber experiments showed that OSS reduced KLF4 transcription and increased BMP6 expression, whereas LSS had the opposite effect. Moreover, siRNA‐mediated KLF4 knockdown confirmed its role as a transcriptional repressor of BMP6. These findings provide a mechanistic explanation for the preferential calcification of arterial bifurcations, where OSS is prevalent, and highlight the important role of hemodynamic factors in the regulation of vascular calcification. Notably, the findings demonstrate a mechanistic link between mechanical stimuli and BMP6‐mediated calcification. This KLF4–BMP6 axis represents a novel link between hemodynamic stress and the molecular pathways driving calcification, offering potential strategies to modulate BMP6 expression through flow normalization or KLF4 activation. This study not only established BMP6 as a critical mediator in vascular calcification pathogenesis but also revealed novel mechanistic insights into how hemodynamic forces and molecular cross‐talk coordinate this process. In the future, we anticipate exploring molecules that could serve as mechanosensitive receptors for KLF4–BMP6 signaling axis activation.

The molecular cross‐talk between ECs and VSMCs was also a major finding of this study. The study findings revealed that the ECs cluster exhibited robust interactions with specific VSMCs subsets (SMC3, SMC5, and SMC8) via the BMP signaling pathway, which is particularly active in CAS patients with symptomatic plaques. This study showed that BMP6 significantly enhanced calcium deposition in VSMCs and concomitantly upregulated the expression of osteogenic markers MSX2 and RUNX2. Our data suggested that EC‐derived BMP6 was transported via exosomes to VSMCs, where it directly induced osteogenic differentiation. The discovery of exosome‐mediated BMP6 transfer between ECs and VSMCs introduces a novel paracrine dimension to BMP signaling in vascular pathology. Such cell–cell communication may explain the spatial progression of calcific lesions observed in CAS, where disturbed flow initiates calcification, which subsequently propagates to adjacent areas. While the data highlight EC3–SMC interactions mediated by BMP6, future studies should explore additional cross‐talk mechanisms between other EC subtypes and SMCs. Furthermore, the molecular mechanisms by which BMP6 promotes vascular development were identified. EC‐derived BMP6 interacts with the ACVR1–BMPR2 receptor complex on VSMCs, thereby activating the p‐SMAD1/5/8 signaling pathway to promote calcification. Pharmacological inhibition of BMPR2 using LDN‐214117 or blockade of downstream p‐SMAD1/5/8 signaling with asiaticoside eliminated BMP6‐induced osteogenic differentiation, confirming the centrality of this pathway. Notably, the BMP signaling axis can also activate the MAPK and PI3K/AKT pathways through type I receptor complexes, which synergistically regulate osteogenic gene expression and matrix mineralization.^[^ ^38^ ^]^ Although the results of this study established p‐SMAD1/5/8 as the primary axis, the potential contributions of these alternative pathways, particularly in modulating cellular responses to sustained BMP6 exposure, could not be excluded. Furthermore, feedback mechanisms likely fine‐tune BMP6 signaling intensity. Negative regulators, such as SMAD6/7 and transmembrane pseudoreceptors, attenuate BMP signaling by competing for receptor binding or by disrupting SMAD complex formation.^[^ ^39^ ^]^ The observed differential sensitivity of VSMC subsets to BMP6 in the scRNA‐seq data (Figure 2B,C) may reflect the heterogeneous expression of such modulators. Future studies delineating the spatiotemporal dynamics of these inhibitory components, particularly in response to hemodynamic stress, will elucidate how BMP6‐driven calcification is spatially constrained in atherosclerotic plaques.

Beyond mechanistic exploration, the identification of BMP6 as a key mediator of vascular calcification opens new avenues for pharmacological interventions targeting this pathway. Previous studies reported that Naotaifang attenuates inflammation and ferroptosis via the BMP6/SMAD signaling pathway.^[^ ^40^ ^]^ Furthermore, the small‐molecule inhibitor LDN‐193189 effectively reversed iron overload in mice by competitively binding to BMP receptors and suppressing hepcidin expression.^[^ ^41^ ^]^ Notably, preclinical evidence from the present study demonstrated that the pharmacological inhibition of BMPR2 with LDN‐214117 or the blockade of downstream p‐SMAD1/5/8 signaling with asiaticoside effectively attenuated the osteogenic differentiation of VSMCs, both in in vitro and animal models. These findings highlight the therapeutic potential of BMP6 signaling inhibitors for vascular calcification in CAS. However, the clinical transition is contingent upon overcoming several fundamental barriers. Foremost is the development of therapeutic strategies that can selectively suppress BMP6‐mediated pathological calcification, alongside the need to establish robust longitudinal safety data through systematic preclinical and clinical evaluations to ensure therapeutic viability. Future studies should prioritize the development of tissue‐specific BMP6 inhibitors and rigorously validate their efficacy using large‐scale preclinical animal models, thereby facilitating the clinical translation of this novel therapeutic strategy for attenuating vascular calcification in CAS.

Despite the significant findings of this study, it has some limitations. First, the exosome‐mediated BMP6 transfer hypothesis requires in vivo validation using lineage‐tracing models or exosome‐specific knockdown approaches. Second, although some understanding of the BMP6‐related signaling pathway has been gained, specific inhibitors or modulators targeting the BMP6 or p‐SMAD signaling pathways require further development and optimization. Finally, this study focused primarily on the role of BMP6 in vascular calcification in CAS. Future research could expand to other types of vascular diseases to comprehensively evaluate the role of BMP6, employ multi‐omics approaches to map the BMP6 interactome, and identify coregulatory networks.

The present study identified the crucial role of BMP6 in CAS and elucidated its mechanism of downstream vascular calcification induced by the EC–VSMC interaction under hemodynamics. This study provides a novel potential target for the treatment of calcification in CAS.

## 4 Experimental Section

### Patients

Carotid artery plaques were collected from 12 patients with CAS undergoing carotid endarterectomy (CEA). This study was performed in accordance with the guidelines of the Declaration of Helsinki. All the participants or their legal proxies provided written informed consent before participating in the study. This study was approved by the Ethics Committee of the First Affiliated Hospital of Zhengzhou University (2020‐KY‐0067‐001).

### scRNA‐seq Library Construction and Sequencing

scRNA‐seq libraries were prepared using the SeekOne MM Single Cell 3′ library preparation kit. Briefly, an appropriate number of cells were loaded into the flow channel of the SeekOne MM chip, which has 170000 microwells; the cells were allowed to settle in the microwells by gravity. After removing the unsettled cells, sufficient cell‐barcoded magnetic beads (CBBs) were pipetted into the flow channel and allowed to settle in the microwells under a magnetic field. Next, excess CBBs were rinsed out, and the cells in the MM chip were lysed to release RNA, which was captured by the CBB in the same microwell. Subsequently, all CBBs were collected, and reverse transcription was performed at 37 °C for 30 min to label the complementary DNA (cDNA) with cell barcode on the beads. Exonuclease I treatment was performed to remove unused primers from the CBBs. Subsequently, the barcoded cDNA on the CBBs was hybridized with a random primer containing the reads 2 SeqPrimer sequence on the 5′ end to generate second‐strand DNA with the cell barcode on the 3′ end. The resulting second‐strand DNA was denatured from the CBBs, purified, and amplified using PCR. The amplified cDNA product was cleaned to remove unwanted fragments and added to a full‐length sequencing adapter and sample index using indexed PCR. The indexed sequencing libraries were cleaned with solid phase reversible immobilization beads, quantified using quantitative PCR (KAPA Biosystems KK4824), and sequenced on an Illumina NovaSeq 6000 with a PE150 read length or a DNBSEQ‐T7 platform with a PE100 read length.

### Analysis of scRNA‐seq Data of Atherosclerotic Plaques

The scRNA‐seq data were analyzed using R version 4.02 as follows: 1) Seurat R package was used to convert scRNA‐seq data as a Seurat object and the “FindVariableFeatures” function was used to select the highly variable genes after quality control; 2) principal component analysis was performed based on the selected variable genes to analyze the scRNA‐seq data; 3) Merge R package was used to integrate single‐cell data, and the “Merge” function was applied in the Seurat object to integrated scRNA‐seq data from 12 atherosclerotic plaques; 4) after integration, uniform manifold approximation and projection (UMAP) were applied to analyze the scRNA‐seq data; 5) Cell types (e.g., endothelial cells, smooth muscle cells, macrophages) were annotated using CellMarker and PanglaoDB dataset, as well as previous studies. Gene expression was visualized using a violin plot, feature plot, dot plot, and heatmap, generated with the Seurat function. Markers for a specific cluster against all remaining cells were identified using the “FindAllMarkers” function (only.pos = TRUE, min.pct = 0.25).

GO enrichment analysis of the marker genes was performed using the Cluster Profiler R package, correcting for gene length bias. GO terms with corrected *p*‐values <0.05 were considered significantly enriched. The KEGG was a database resource for understanding high‐level functions and utilities of biological systems, such as cells, organisms, and ecosystems, from molecular‐level information, especially large‐scale molecular datasets generated using genome sequencing and other high‐throughput experimental technologies. The cluster Profiler R package was used to test the statistical enrichment with a corrected *p*‐value <0.05 of marker genes in the KEGG pathways. Protein interactions of marker genes with a modulus of logFC of >1 and a corrected *p*‐value <0.05 were obtained and visualized using Cytoscape.

### Cell Culture and Co‐Culture

Mice VSMCs were purchased from Procell Life Science & Technology (CP‐M076) and grown in Dulbecco's modified Eagle medium (DMEM, Gibco)—supplemented with 10% fetal bovine serum (FBS), penicillin (10 U mL^−1^), and streptomycin (10 µg mL^−1^)—; it was used between passages 1 and 6. HUVECs were purchased from Shanghai Zhong Qiao Xin Zhou Biotechnology Co., Ltd. (DFSC‐EC‐01) and grown on gelatin‐coated dishes in an EC growth medium (Gibco, Thermo Fisher Scientific)—supplemented with SingleQuots (Lonza), penicillin (10 U mL^−1^), and streptomycin (10 µg mL^−1^)—; it was used between passages 1 and 6. Cells were maintained in a humid incubator at 37 °C with 5% CO_2_ and air. To induce osteogenic differentiation, the medium was changed to DMEM, with 4.5 g L^−1^ glucose, 10% FBS, GlutaMAX supplement, penicillin/streptomycin (control medium), 10 nmol L^−1^ dexamethasone, and 10 mmol L^−1^ β‐glycerophosphate, and 100 µmol L^−1^ L‐ascorbic acid 2‐phosphate added to the medium (osteogenic medium, OM). VSMCs were cultured for 3 weeks in OM, and the medium was changed every 2 or 3 days. To examine the impact of BMP6 (PeproTech, London, UK) on the osteogenic differentiation of VSMCs in vitro, cells were exposed to different concentrations of human BMP6 (0, 30, and 50 ng mL^−1^) and cultured for 4 days.

Co‐cultures were established by plating HUVECs and HAoSMCs (purchased from Procell Life Science & Technology, CP‐H081). HUVECs in the lower chamber were pretreated with or without enhanced BMP6 in six‐well Transwell units, whereas HAoSMCs were seeded into the well inserts of six‐well plates with a 0.4‐µm pore‐sized filter (Becton Dickinson). HUVECs and HAoSMCs were maintained in medium 199 or F12 Ham Kaighn's modification (F12K), respectively, supplemented with 2% FBS until the cells were fully attached to the membrane. BMP6‐enhanced HUVECs in well inserts were pretreated with or without GW4869 (Sigma–Aldrich), which was a pharmacological compound known to effectively inhibit exosomes biogenesis or release, and co‐cultured with HAoSMCs in a complete F12K medium for 48 h.

### Flow Apparatus for EC Culture

The equipment for simulating LSS and OSS in vitro was purchased from Shanghai Naturethink Life & Scientific Co., Ltd. The equipment consisted of a peristaltic pump, smoothing device, flow chamber, and liquid storage bottle connected to a silicone hose in a closed loop. Cultured ECs were grown on gelatin‐coated slides and placed in a parallel‐plate flow chamber connected to a perfusion loop system. The flow channel in the chamber was created using a silicon gasket that was 2.496 cm wide (w), 5.725 cm long, and 0.025 cm high (h). The shear stress (τ) generated on the ECs was estimated as 6Qµ wh^−2^, where Q is the flow rate and µ is the perfusate viscosity. ECs were exposed to LSS (12 ± 4 dynes cm^−2^) or OSS (0.5 ± 4 dynes cm^−2^). The RNA and protein were extracted from ECs maintained in a humid incubator with 5% CO_2_ and air at 37 °C up to 24 h.

### Exosome Isolation and Identification

The co‐culture medium was transferred to 50‐mL centrifuge tubes and centrifuged at 300 g for 10 min to collect the supernatant. Subsequently, the supernatant was subjected to low‐speed centrifugation (3000 × g for 10 min) to remove cell debris. The supernatant was subjected to centrifugation at 10000 × g for 30 min, followed by ultracentrifugation at 100000 × g for 120 min (Optima L‐100XP; Beckman Coulter, USA). The pelleted exosomes were washed twice with 1 mL PBS and centrifuged at 100000 × g for 120 min. The exosome‐enriched pellet was resuspended in 100 µL of PBS. Exosomal proteins were quantified using a bicinchoninic acid (BCA) kit (Beyotime Biotechnology, Shanghai, China). To identify the isolated exosomes, a Hitachi H‐7650 transmission electron microscope (Hitachi, Tokyo, Japan) was used to observe their morphology, and a molecular size analyzer (ZetaView PMX 110, Particle Metrix, Germany) was used to measure their size distribution. Western blot analysis was conducted to detect the expression of the exosomal surface marker proteins CD9, CD63, and TSG101.

### ALP Activity

ALP activity was measured as described previously.^[^ ^42^ ^]^ Briefly, the VSMCs were washed with PBS and scraped into a solution. Spectrophotometric measurement of p‐nitrophenol released at 37 °C was utilized to analyze ALP activity. ALP activity was normalized to the total protein content of the cell lysates.

### Alizarin Red Staining

Alizarin Red staining was used to assess calcium deposition in the cells. Cells were washed in PBS, fixed in 4% paraformaldehyde for 15 min, followed by rinsing in distilled water, and stained with filtered Alizarin Red S solution (#8678, ScienCell) for 5 min. The solution was removed, and images were captured immediately under a microscope. Representative images were selected to represent the mean values for each condition.

### Experimental Animals

All animal experiments were conducted in accordance with the ARRIVE guidelines and approved by the Animal Ethics Committee of the First Affiliated Hospital of Zhengzhou University (2020‐KY‐0067‐001). All the animals used in the present study were male to minimize the effect of estrogen on calcification. For all apolipoprotein E‐deficient (ApoE^−/−^) mice, 8‐week‐old male mice were fed a Western diet (Dyets Company, No. ASHF4; 40% kcal from fat, 1.25% cholesterol) for 7 or 8 weeks. All animals were maintained at a constant temperature (21 ± 1 °C) under a 12‐h light/dark cycle and had free access to water and standard chow.

Eight‐week‐old male ApoE^−/−^ mice were purchased from Spelford (Beijing, China). AAV9‐mediated gene delivery under the control of the endothelial‐specific Cdh5 promoter. The viral vectors (AAV‐Cdh5‐Vector and AAV‐Cdh5‐BMP6) were obtained from OBiO (Shanghai, China) at concentrations of 1.44 × 10^−13^ and 1.56 × 10^−13^vg mL^−1^, respectively. Furthermore, for endothelial‐selective KLF4 knockdown, ApoE^−/−^ mice received tail vein injections of AAV9 vectors encoding a short hairpin RNA against KLF4 (shKLF4), driven by the endothelial‐specific mouse Cdh5 promoter (AAV9‐Cdh5‐shKLF4). ≈1 week after gene delivery via tail vein injection, the mice were anesthetized and subjected to PCL. Briefly, three of the four caudal branches of the left carotid arteries (left external carotid, internal carotid, and occipital arteries) were ligated using a 6‐0 silk suture (HEAD [BEIJING] BIOTECHNOLOGY Co., Ltd., SUT‐S104), whereas the superior thyroid artery was left intact.^[^ ^43^, ^44^, ^45^ ^]^ The right carotid arteries were used as controls. The mice were fed a Western diet immediately after surgery. Eight weeks after ligation, the mice were sacrificed and fixed for 5 min by perfusion through the left cardiac ventricle with 4% paraformaldehyde in a PBS buffer under physiological pressure. Ligated carotid arteries were harvested and subjected to histological analysis and immunostaining.

BMP6‐flox mice (S‐CKO‐01436) were purchased from Cyagen (Madison, WI, USA). EC‐specific BMP6 knockdown mice (BMP6*^ECKD^*; i.e., Cdh5*^Cre^*; BMP6*^fl/fl^*) were generated by breeding male Cdh5*^Cre^* mice with female BMP6*^fl/fl^* mice and crossing their offspring with female BMP6*^fl^*/*^fl^* mice. The generated BMP6*^ECKD^* mice were genotyped and crossed with ApoE^−/−^ mice to generate Cdh5*^Cre^*, BMP6*^fl/fl^*, and ApoE^−/−^ (BMP6*^ECKO^* ApoE**^−/−^**) mice. Littermate wild‐type mice (BMP6*^fl/fl^* ApoE^−/−^) were used as controls. Eight‐week‐old male BMP6*^ECKO^* ApoE**^−/‐^** and littermate BMP6*^fl/fl^* ApoE^−/‐^ mice were subjected to PCL and fed a Western diet. Eight weeks after ligation, the mice were sacrificed and fixed for 5 min by perfusion through the left cardiac ventricle with 4% paraformaldehyde in a PBS buffer under physiological pressure. Ligated carotid arteries were harvested and subjected to histological analysis and immunostaining. Mouse genotypes were determined using a one‐step mouse genotyping kit (PD101‐01; Vazyme).

### RNA Isolation and RT‐qPCR

Total RNA from cultured cells or harvested carotid artery tissue was extracted using TRIzol reagent (Vazyme, RC112‐01). RNA was reverse transcribed to cDNA using a reverse transcription kit (Vazyme, R333‐01). Briefly, cDNA synthesis was performed by incubating the samples for 30 min at 37 °C for reverse‐transcription, followed by 5 s at 85 °C for inactivating the reverse transcriptase using heat. For detecting the interested genes, 0.25 µm of primers, 0.5 µL of cDNA with 2 × SYBR Green qPCR Master Mix (Vazyme, Q712‐02) were added to a 96‐well plate for qPCR analysis (Bio‐Rad, Shanghai, China). A standard thermocycling protocol (95 °C for 5 s and 60 °C for 30 s; total: 40 cycles) was used to amplify the gene copy number. Relative expression was calculated using the 2^−△△Ct^ method, with normalization to actin expression. The primer sequences (Beijing Qingke Biotechnology Co., LTD) used in this study are provided in Table S2 (Supporting Information).

### Western Blot Analysis

RIPA (strong) protein lysate supplemented with protease and phosphatase inhibitors was added to the cells and CAS tissues. The samples were placed on ice for 30 min and centrifuged at 12000 g for 10 min. The supernatant containing the proteins was collected. The BCA method was used to detect the protein concentration, which was calculated based on the final protein standard curve. The loading buffer was added five times to the samples, which were then boiled at 100 °C for 10 min. Proteins of different molecular weights and pre‐stained protein marker 26616 (Thermo Fisher Scientific) were separated using sodium dodecyl sulfate‐polyacrylamide gel electrophoresis. After transferring the protein to 0.2‐µm polyvinylidene fluoride membranes (Millipore, USA, Cat. ISEQ00010), the membranes were blocked in 5% skimmed milk powder dissolved in a 1 × Tris‐buffered saline with Tween 20 (TBST) buffer at room temperature for 1 h, followed by incubation with the primary antibody diluted using 5% skimmed milk powder at 4 °C overnight. The membranes were washed thrice in TBST and incubated with the corresponding secondary antibodies at a dilution of 1:10000 for 1.5 h at room temperature. Finally, the membranes were washed thrice with TBST and visualized using enhanced chemiluminescence (Cell Signaling Technology, 39864S). The protein bands were visualized using the Bio‐Rad ChemiDoc Imaging System. The primary antibodies used in this study are listed in Table S3 (Supporting Information).

### Immunofluorescence and Immunohistochemistry Staining

HAoSMCs, human aortic plaques, and mouse carotid artery sections were fixed with 4% paraformaldehyde and then permeabilized with 0.1% Triton X‐100. The cell samples were blocked with 5% bovine serum albumin (BSA) at room temperature for 2 h. Subsequently, they were incubated with the primary antibody overnight at 4 °C. The membranes were then incubated with Alexa Fluor secondary antibodies for 2 h at room temperature in the dark. Nuclei were stained with 4′,6‐diamidino‐2‐phenylindole (Invitrogen) in PBS for 5 min. Images were acquired using a FluoView FV1000 laser scanning confocal microscope (Olympus, Tokyo, Japan). To verify the specificity of the antibodies used in this study, isotype controls from the same host species were used at the same working concentrations under the same conditions as those used for the primary antibodies. To distinguish the authentic target staining from the background, a negative control containing only a secondary antibody was used in each experiment. Immunofluorescence staining was quantified using the ImageJ software (version 1.53, National Institute of Health, USA). Representative images were selected to represent the mean values for each condition.

The mice were anesthetized and then perfused successively with normal saline and 4% paraformaldehyde. The carotid arteries were excised, infiltrated with paraffin, and cut into 4‐µm‐thick continuous sections. After dewaxing and rehydrating the sections, they were immersed in an antigen retrieval solution (80 mm citric acid, 20 mm sodium citrate, and pH 6.0) at 94 °C for 20 min. The sections were incubated with 3% hydrogen peroxide for 10 min to eliminate endogenous peroxidase activity. The sections were blocked in PBS containing 1% BSA and 0.3% TX‐100 for 30 min and then incubated with the primary antibody overnight at 4 °C. The sections were washed three times in PBS containing 0.1% TX‐100. For immunohistochemistry, a Histostain‐SP kit (Invitrogen) was used to generate signals.

### Chromatin Immunoprecipitation (ChIP) and ChIP‐seq

ChIP assays were performed using a ChIP Assay Kit (Cat#10086; Millipore). ECs cultured in 10‐cm plates were fixed with 243 µL of 37% paraformaldehyde (Electron Microscopy Sciences, 15714) and cross‐linked for 15 min at room temperature. Cross‐linking was stopped by adding 2.5 m glycine until a final concentration of 0.125 m was reached. The cells were then lysed and sonicated to shear the chromatin into fragments of 200–1000 bp, and the fragment length was confirmed by gel electrophoresis. Subsequently, immunoprecipitation was conducted by incubation with a primary antibody (anti‐KLF, Proteintech, 11880‐1‐AP) in combination with protein A/G‐conjugated magnetic beads overnight at 4 °C with rotation. Protein‐DNA cross‐links were reversed, and the DNA was purified to remove chromatin proteins, followed by qRT‐PCR using specific primers (forward: AGCATCTGCACTGAGGAAATAG and reverse: GCTCTCTGACCTAGCTTCATTC).

### Statistical Analysis

Statistical analyses were performed using GraphPad Prism 8 software. All data were tested for normality and variance. If significant, the Student's *t*‐test was used to compare two groups, or one‐way analysis of variance followed by Tukey's post‐hoc test was used to compare more than two groups. The data were presented as the means ± SDs. The sample size for each statistical analysis was indicated in the corresponding Figure legend. Statistical significance was set at a *p*‐value of <0.05.

### Ethics Approval and Consent to Participate

This study was performed in accordance with the guidelines of the Declaration of Helsinki. All the participants or their legal proxies provided written informed consent before participating in the study. All animal experiments were conducted in accordance with ARRIVE guidelines. All studies were approved by the Ethics Committee of the First Affiliated Hospital of Zhengzhou University (2020‐KY‐0067‐001).

### Patient Consent Statement

All the participants or their legal proxies provided written informed consent before participating in the study.

## Conflict of Interest

The authors declare no conflict of interest.

## Author Contributions

SL, SC, and PL contributed to the study design and manuscript drafting. FZ, GR, XW, JZ, CL, and YG contributed to the data acquisition, analysis, and interpretation. YX, ZX, YW and JX made substantial contributions to conception and design. All the authors have read and approved the final version of this manuscript.

## Supporting information



Supporting Information

Supporting Information

## Data Availability

Our data are available in a public, open access repository. All raw scRNA‐seq data are available through the Gene Expression Omnibus under accession number GSE309462 and the link is https://www.ncbi.nlm.nih.gov/geo/query/acc.cgi?acc = GSE309462.
