# Supplementary material for: Endothelial BMP6 Drives Hemodynamic‐Dependent VSMCs Calcification in Carotid Atherosclerosis
Source: Adv Sci (Weinh). 2025 Oct 13;13(2):e02801. doi: 10.1002/advs.202502801 (PMC12786358; doi:10.1002/advs.202502801)
Supplement: Supplementary file 1 — Supporting Information [file ADVS-13-e02801-s001.docx]

Supporting Information

**Endothelial BMP6 drives hemodynamic-dependent VSMCs calcification in carotid atherosclerosis**

*Shen Li, Shuang Cao, Peipei Li, Feng Zhang, Gangfeng Ren, Xin Wang, Jiawei Zhao, Chen Liu, Yuan Gao, Jie Xu*, Yongjun Wan*, Zongping Xia*, and Yuming Xu**


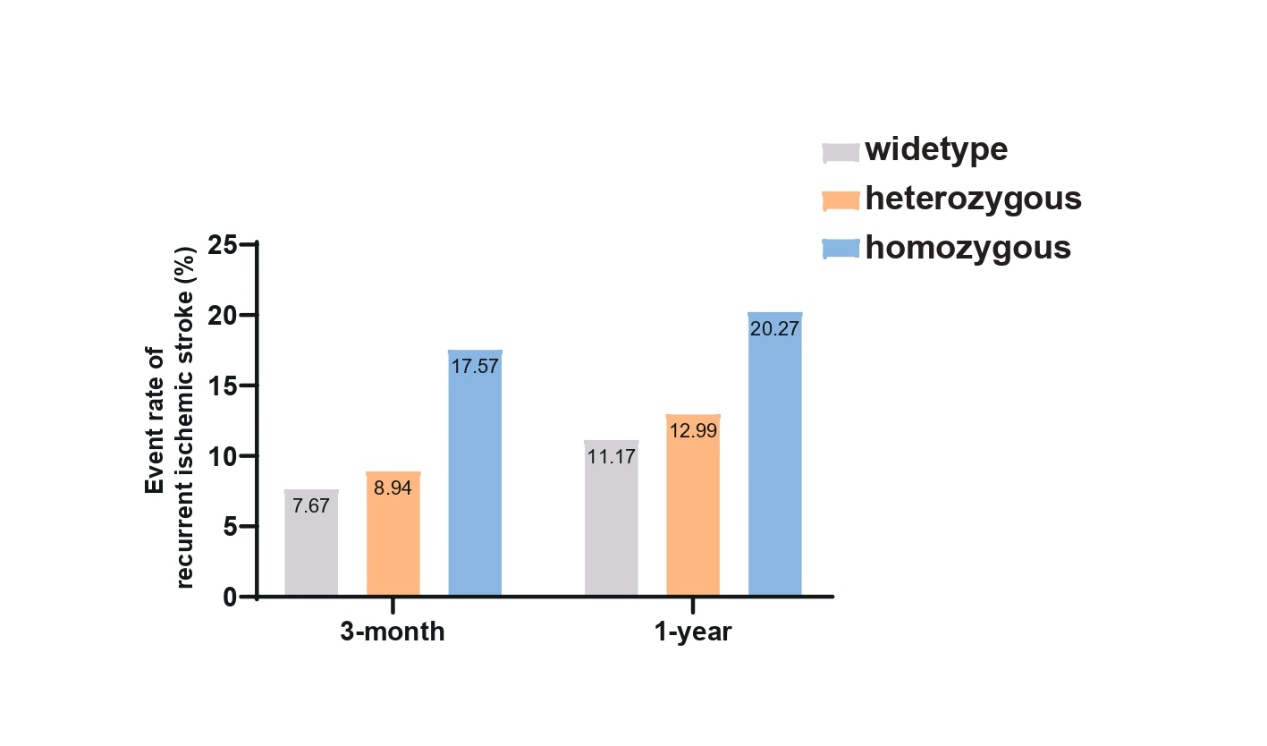


**Figure S1.** Bar chart depicting the event rates of recurrent ischemic stroke (%) in different BMP6 mutation groups at 3-month and 1-year follow-up. The homozygous (blue) genotype consistently shows the highest recurrence rates compared with the wildtype (gray) and heterozygous (orange) groups (17.57% vs. 7.67% and 8.94% at 3 months, 20.27% vs. 11.17% and 12.99% at 1 year).


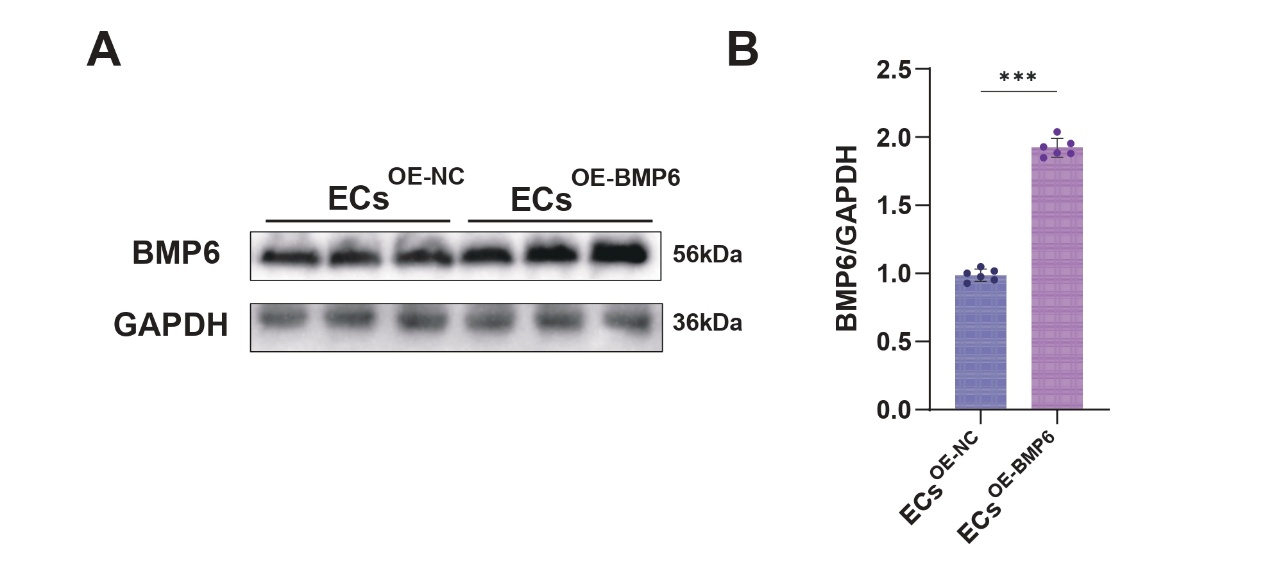


**Figure S2.** Validation of BMP6 overexpression in endothelial cells (ECs). (A) Western blot analysis depicting BMP6 protein expression in the ECs^OE-NC^ and ECs^OE-BMP6^ groups. GAPDH was used as a loading control. (B) Quantitative analysis of BMP6 protein expression (n=6). Data are presented as mean ± standard deviation. *P* values were determined using the unpaired two-tailed Student’s t-test; ****P*<0.001. BMP, bone morphogenic protein; GAPDH, glyceraldehyde-3-phosphate dehydrogenase.

**
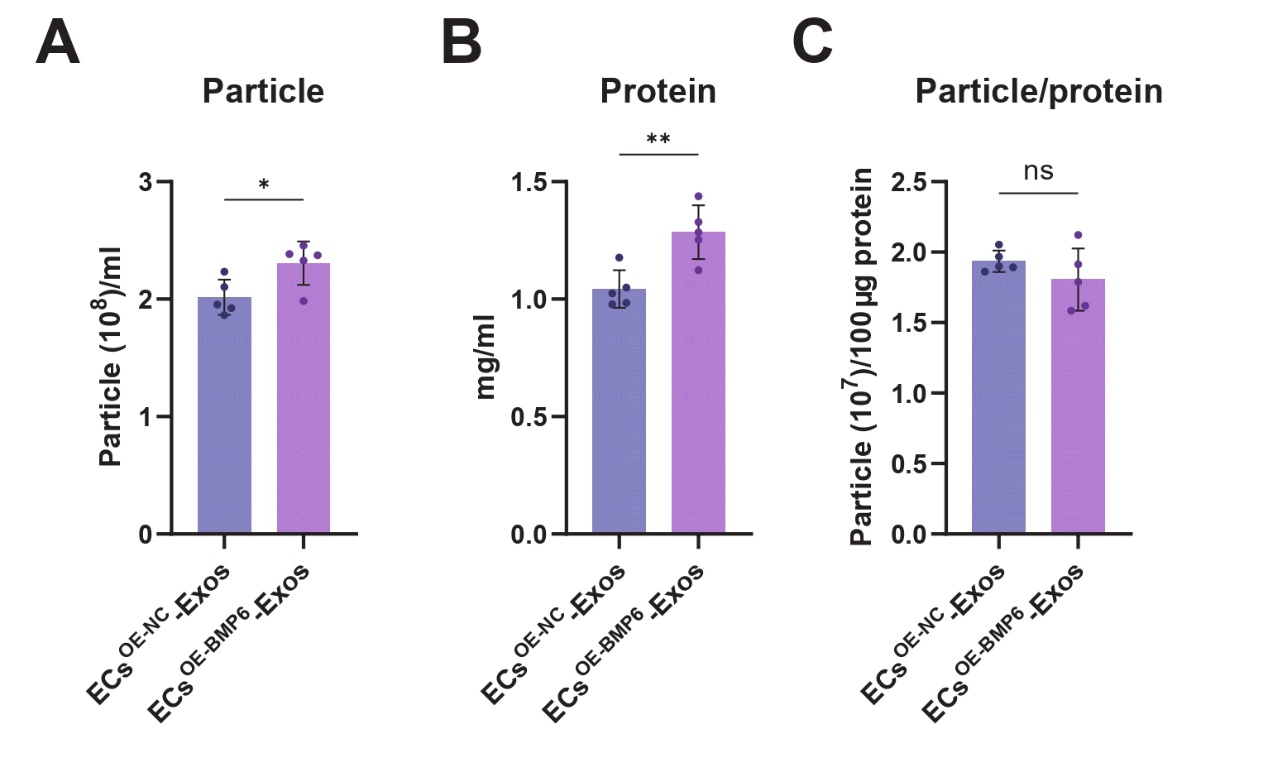
**

**Figure S3.** Particle and protein yield of ECs^OE-NC^-Exos and ECs^OE-BMP6^-Exos. (A) Number of particles isolated per milliliter in ECs^OE-NC^-Exos and ECs^OE-BMP6^-Exos (n=5). (B) The protein yield per milliliter in ECs^OE-NC^-Exos and ECs^OE-BMP6^-Exos (n=5). (C) Amount of particles per 100μg protein in ECs^OE-NC^-Exos and ECs^OE-BMP6^-Exos (n=5). Data are presented as mean ± standard deviation. *P* values were determined using the unpaired two-tailed Student’s t-test; ns, no significant difference, **P*<0.05 and ***P*<0.01.


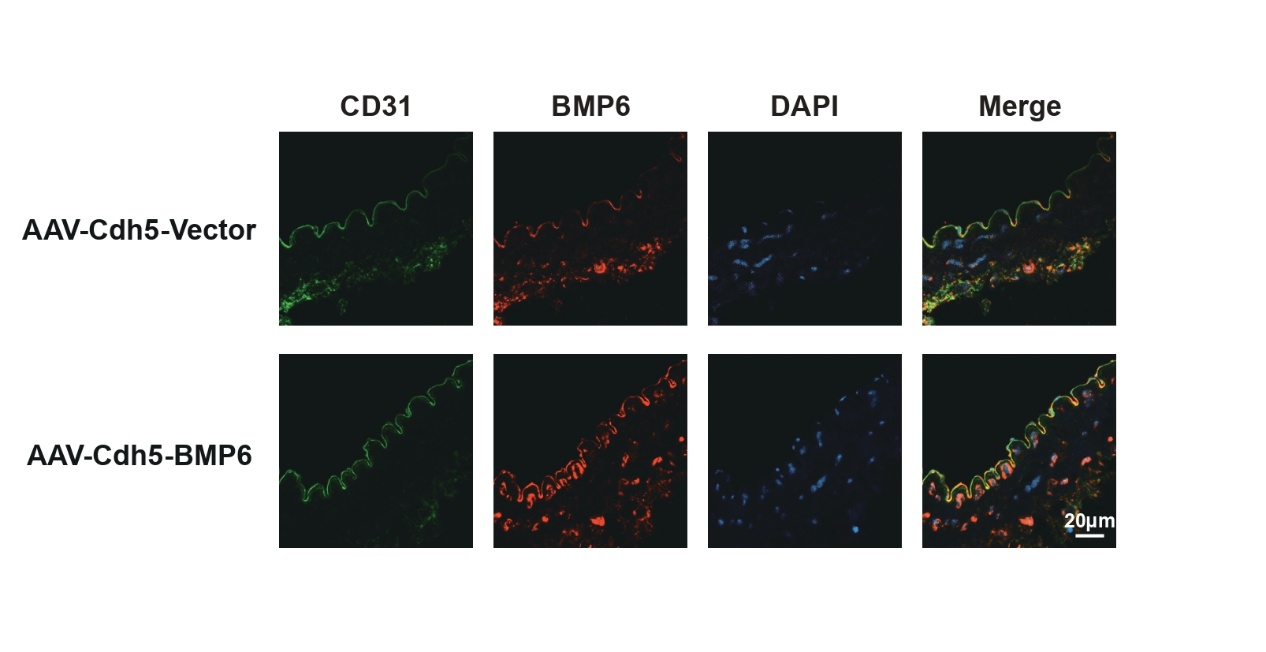


**Figure S4.** Validation of AAV-Cdh5-BMP6 mice. Immunofluorescence staining of carotid arteries from mice injected with AAV-Cdh5-Vector or AAV-Cdh5-BMP6. Endothelial cells (ECs) were labeled with CD31 (green), BMP6 protein was visualized (red), and nuclei were stained with DAPI (blue). Merged images illustrate co-localization. Noticeably enhanced BMP6 expression is observed in CD31⁺ ECs of the AAV-Cdh5-BMP6 group. Scale bar=20 μm.


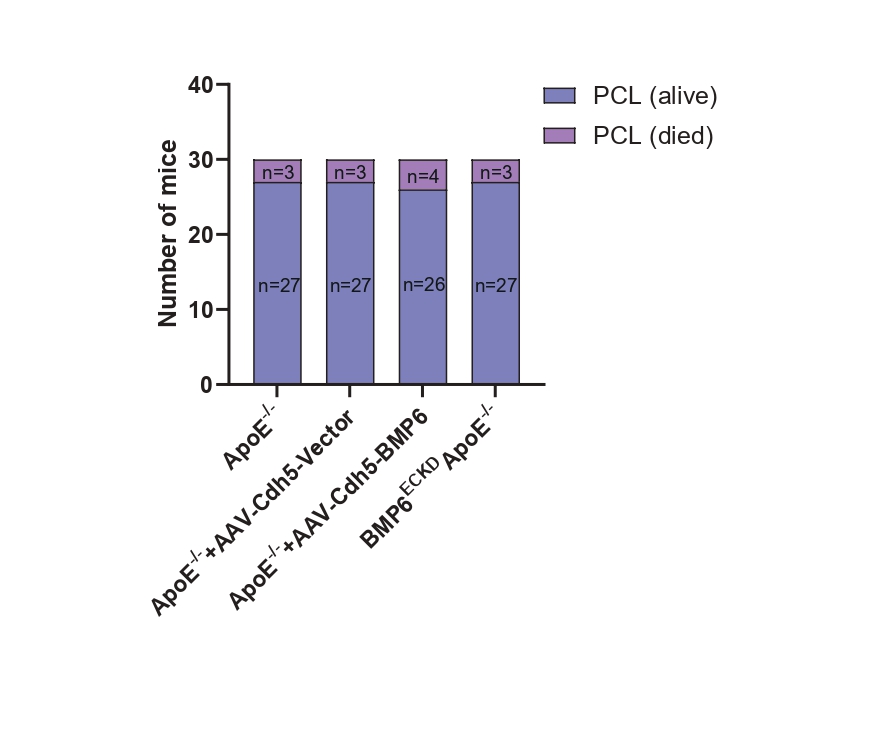


**Figure S5.** Survival assessment of mice following partial carotid ligation (PCL) in the ApoE^-/-^, ApoE^-/-^+ AAV-Cdh5-Vector, ApoE^-/-^ + AAV-Cdh5-BMP6, and BMP6^ECKD^ ApoE^-/-^ groups. Following PCL, the number of surviving (blue) and deceased (purple) mice was recorded (total n=30).


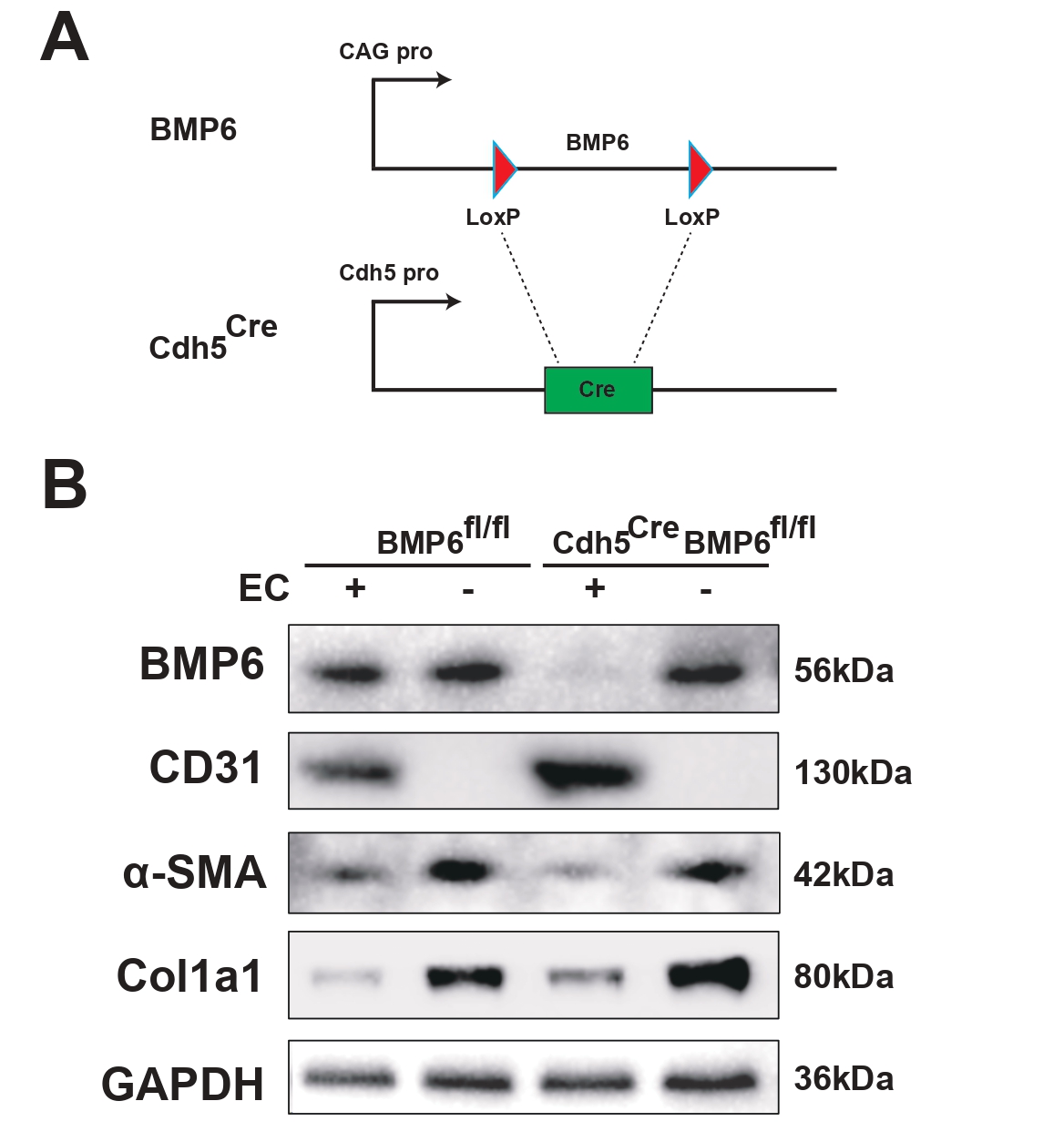


**Figure S6.** Generation and validation of EC-specific BMP6 knockout mice. (A) Schematic diagram showing the breeding approach to generate EC-specific BMP6 knockout mice. (B) Western blots showing a marked reduction of BMP6 protein in ECs of mice. EC, endothelial cell; BMP, bone morphogenic protein; α-SMA, α-smooth muscle actin; Col1a1, collagen type I alpha 1; GAPDH, glyceraldehyde-3-phosphate dehydrogenase.

**
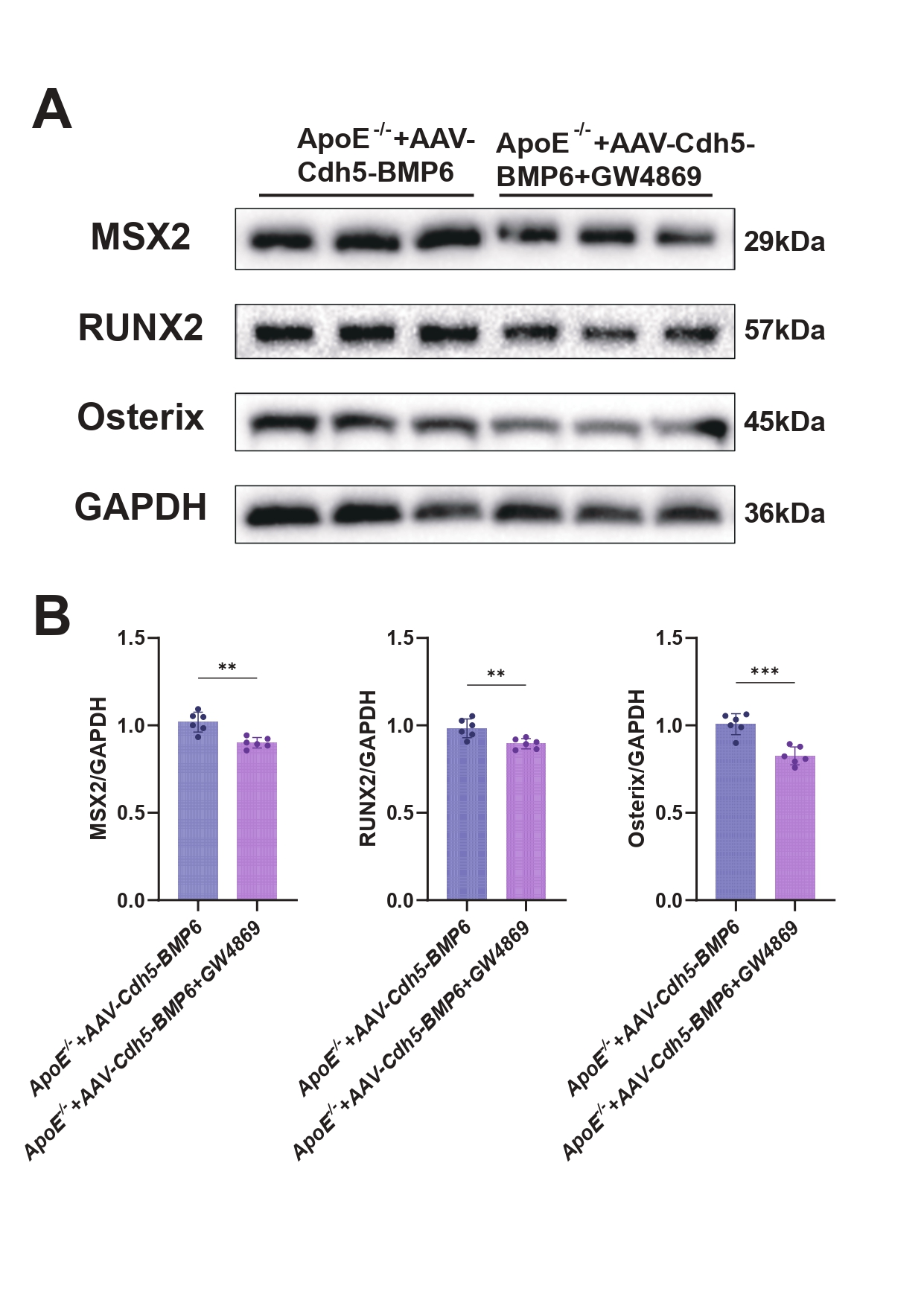
**

**Figure S7.** Effect of GW4869 on osteogenic marker expression in ApoE^-/-^+AAV-Cdh5-BMP6 mice. (A) Western blot analysis of MSX2, RUNX2, and Osterix expression in ApoE^-/-^+AAV-Cdh5-BMP6 mice treated with or without GW4869. GAPDH was used as a loading control. (B) Quantitative data of these proteins are presented (n=6). Data are presented as mean ± standard deviation. *P* values were determined using the unpaired two-tailed Student’s t-test; ***P*<0.01 and ****P*<0.001. MSX2, Msh Homeobox 2; RUNX2, runt-related transcription factor 2; GAPDH, glyceraldehyde-3-phosphate dehydrogenase.


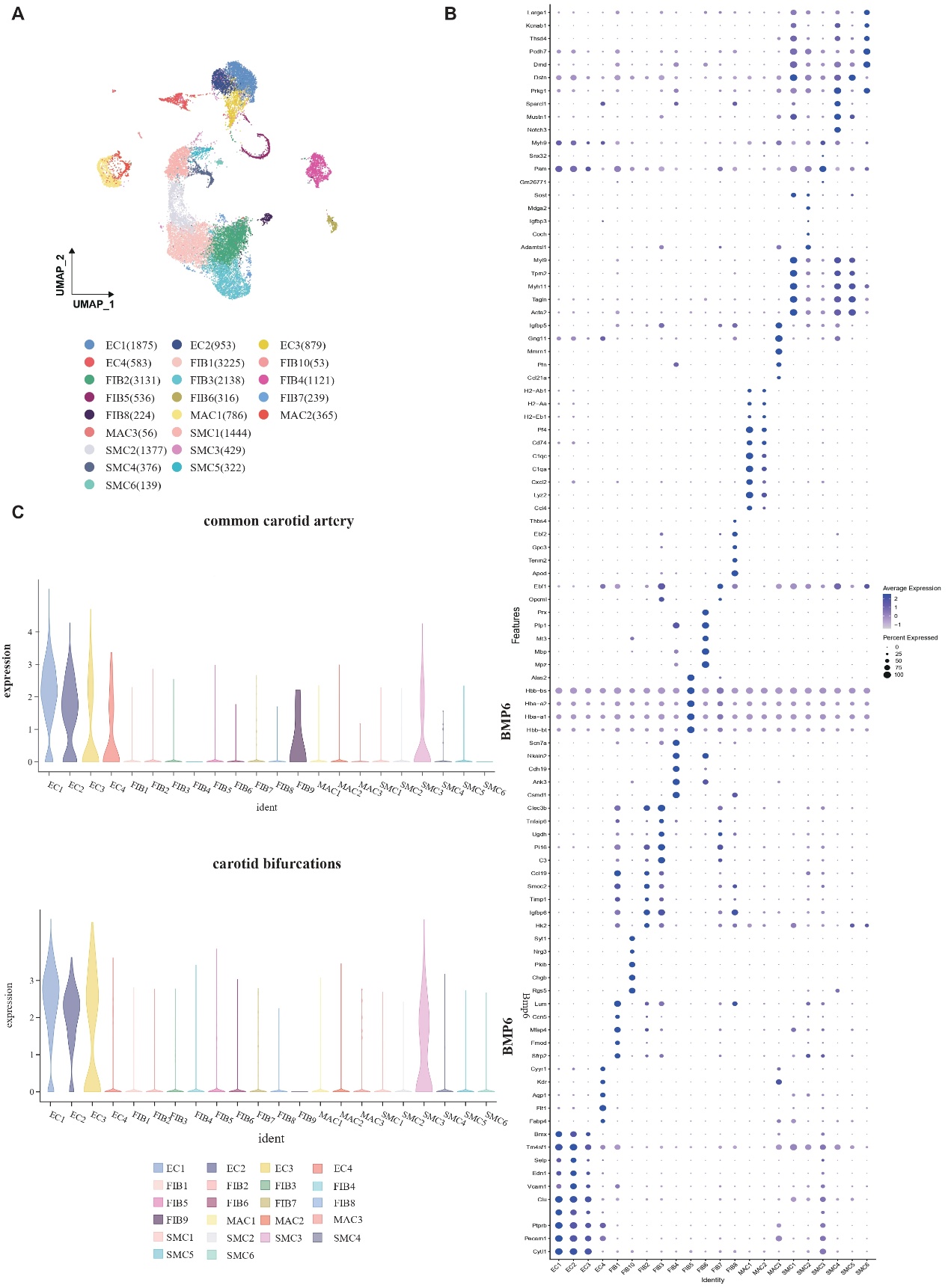


**Figure S8.** Single-cell transcriptional landscape of mouse carotid arteries. (A) Uniform manifold approximation and projection (UMAP) plot illustrating the results of cell annotation following single-cell data clustering, with the number of cells in each cluster shown in parentheses. (B) Dot plot displaying the marker genes used for cell type annotation. Color intensity indicates average expression, and dot size represents the percentage of expressing cells. (C) Split violin plot showing the expression of BMP6 in different cell types in the common carotid artery and carotid bifurcations. BMP, bone morphogenic protein. EC, endothelial cell; FIB, fibroblast; MAC: macrophage; SMC, smooth muscle cell.


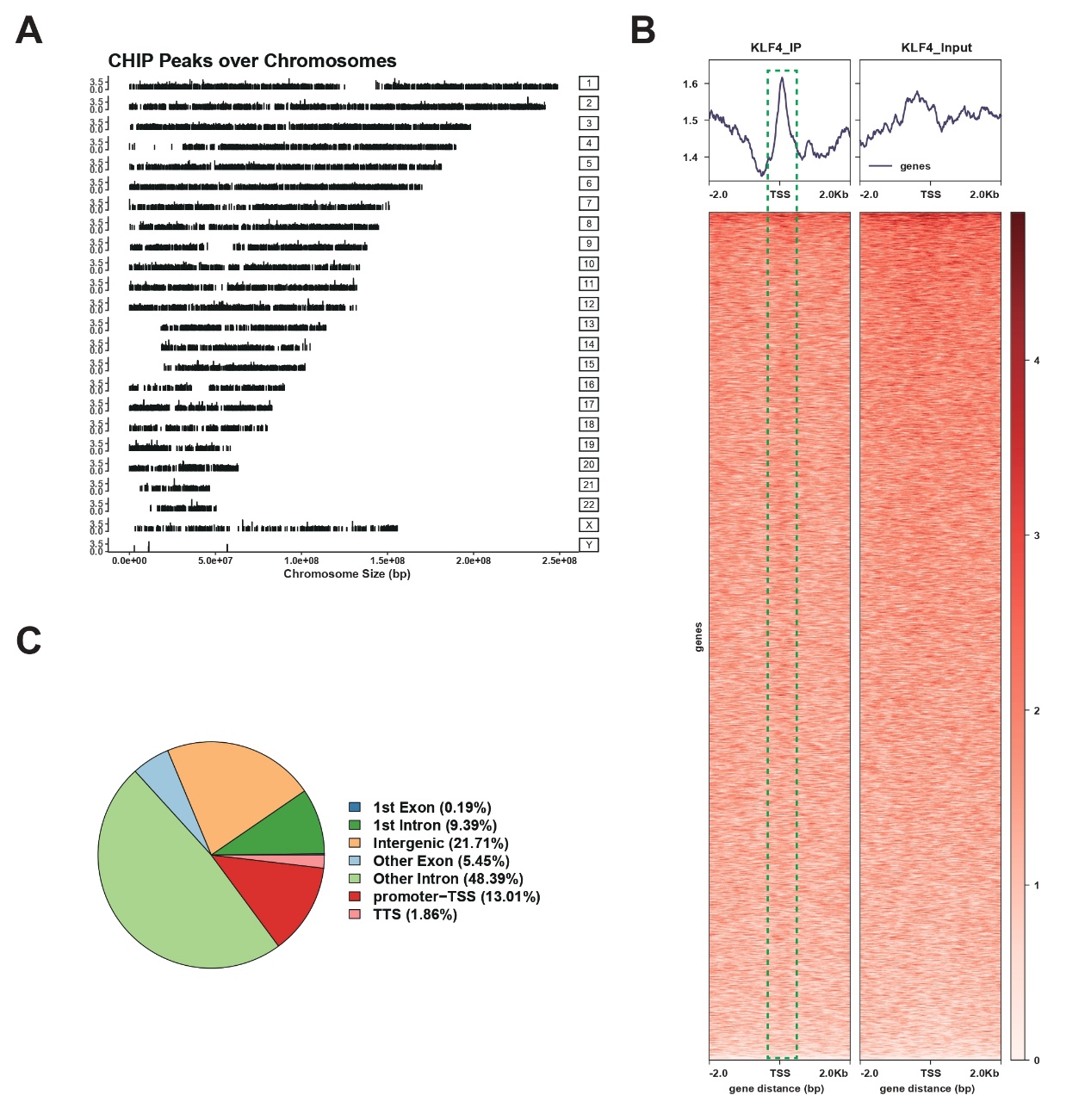


**Figure S9.** Identification of genome-wide DNA binding sites and transcription targets for KLF4. (A) ChIP Peaks over chromosomes assessed using ChIP-seq using the primary antibody against KLF4 (The abscissa represents the length of the chromosome, the right represents the chromosome number, and the left ordinate represents the peak value of each chromosome). (B) Distribution of reads on both sides of the transcription start site (TSS); green dashed box represents the coverage depth of the gene at the corresponding position. (C) Pie diagram shows the ratios of KLF4 binding sites located relative to a transcription unit including intergenic, 1st exon, 1st intron, TTS, promoter, other introns and other exons.


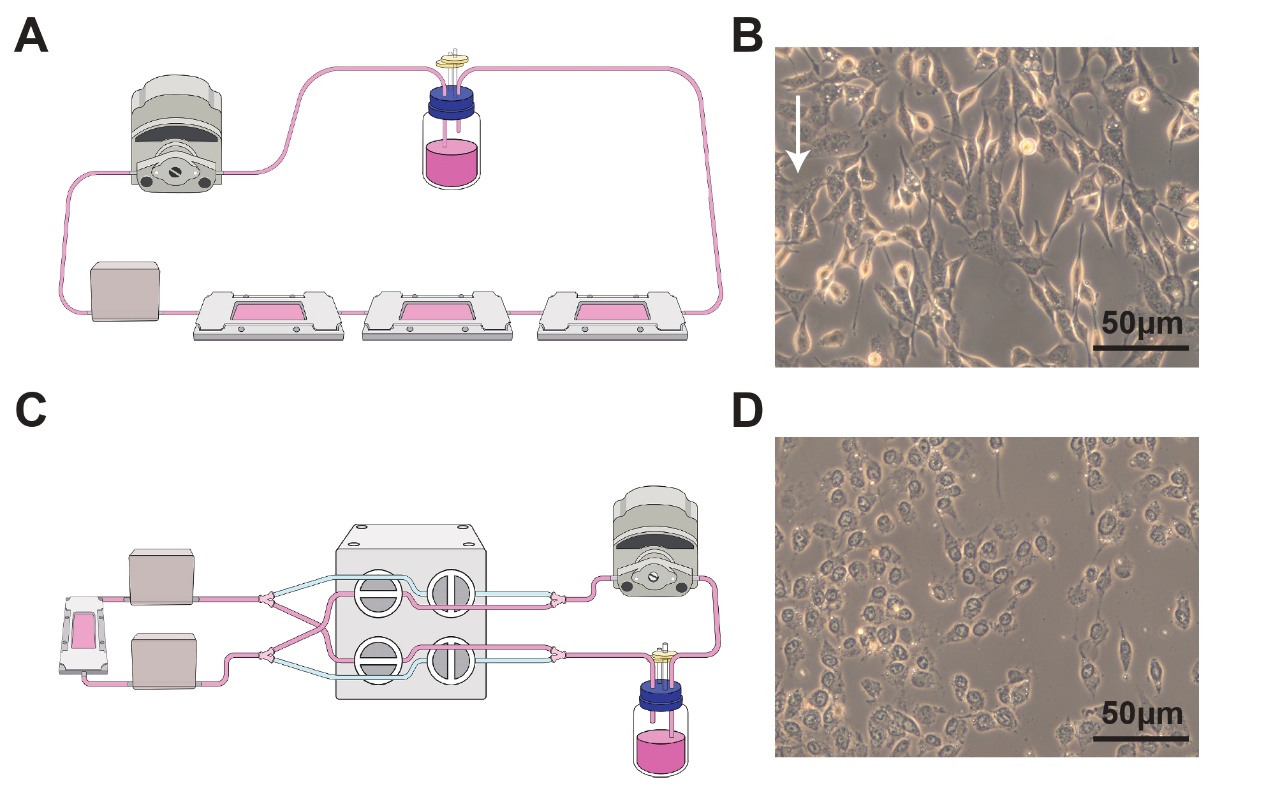


**Figure S10.** Experimental setups for shear stress application and cellular morphology analysis. (A) Schematic of the laminar shear stress (LSS) perfusion system, consisting of a pump, medium reservoir, and parallel flow chambers, to deliver stable unidirectional fluid flow to cultured cells. (B) Microscopic images of human umbilical vein endothelial cells (HUVECs) under LSS (The white arrow indicates the direction of fluid flow), exhibiting an elongated and flow-aligned morphology (scale bar = 50 μm). (C) Schematic of the oscillatory shear stress (OSS) perfusion system, incorporating a flow distributor to generate bidirectional, oscillatory fluid motion. (D) Microscopic images of HUVECs exposed to OSS, displaying a rounded and less polarized phenotype compared to LSS-treated cells (scale bar=50 μm).

**
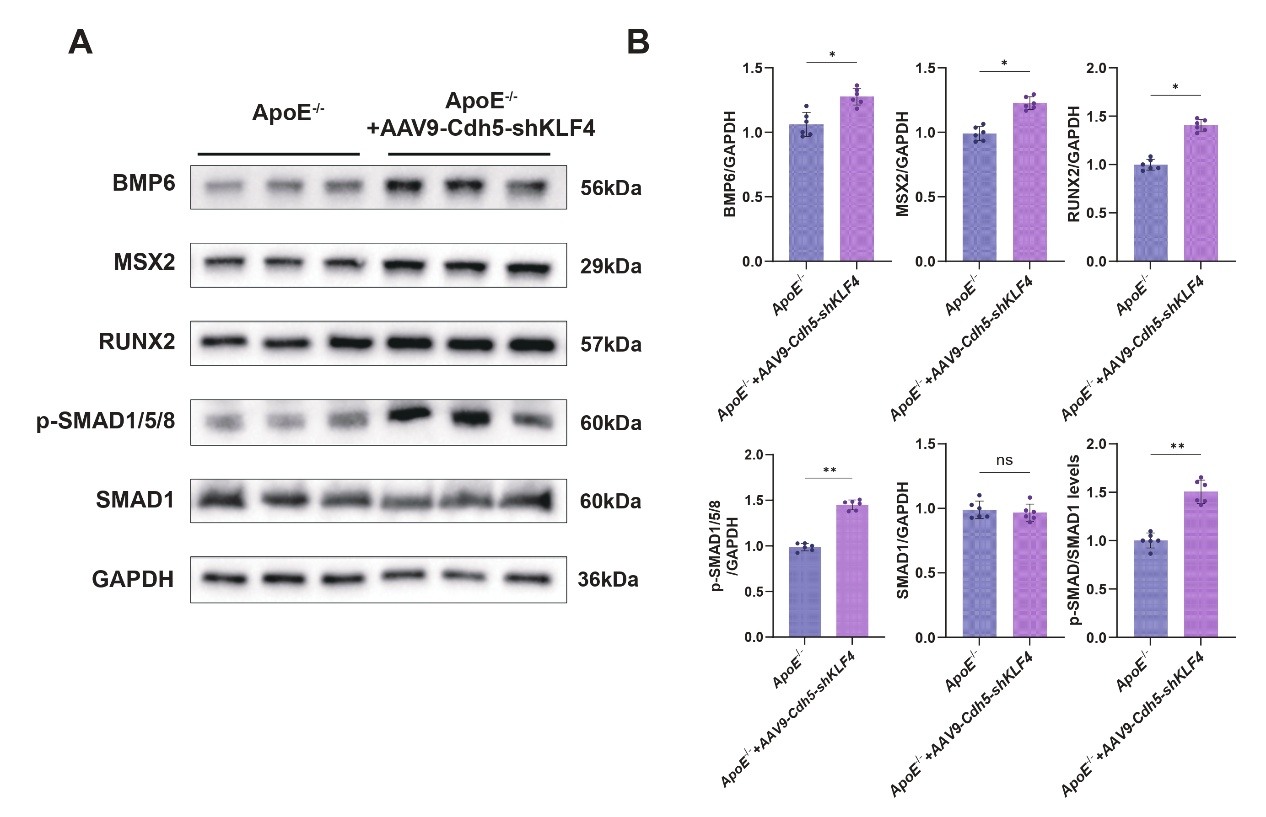
**

**Figure S11.** Protein expression analysis in ApoE^-/-^ and AAV9-Cdh5-shKLF4-treated ApoE^-/-^ mice. (A) Western blot analysis of BMP6, MSX2, RUNX2, p-SMAD1/5/8, and SMAD1 expression in ApoE^-/-^ and ApoE^-/-^+AAV9-Cdh5-shKLF4 mice. GAPDH was used as a loading control. (B) Quantitative data of these proteins are presented (n=6). Data are presented as mean ± standard deviation. *P* values were determined using the unpaired two-tailed Student’s t-test; ns: no significant difference, **P*<0.05 and ***P*<0.01. BMP, bone morphogenic protein; MSX2, Msh Homeobox 2; RUNX2, runt-related transcription factor 2; GAPDH, glyceraldehyde-3-phosphate dehydrogenase.

**Table S1**

**Table S2. Primers for qPCR.**

| **Gene** | **Sequence** |
| --- | --- |
| MSX2(m)-F | CTAAAGGCGGTGACTTGTTTTCG |
| MSX2(m)-R | CGGCTTCTTGTCGGACATGAG |
| RUNX2(m)-F | GACTGTGGTTACCGTCATGGC |
| RUNX2(m)-R | ACTTGGTTTTTCATAACAGCGGA |
| Actin(m)-F | GTGACGTTGACATCCGTAAAGA |
| Actin(m)-R | GCCGGACTCATCGTACTCC |
| KLF4(m)-F | AGGAACTCTCTCACATGAAGCG |
| KLF4(m)-R | GGTCGTTGAACTCCTCGGTC |
| p-SMAD1(m)-F | GCTTCGTGAAGGGTTGGGG |
| p-SMAD1(m)-R | CGGATGAAATAGGATTGTGGGG |
| p-SMAD5(m)-F | TTGTTCAGAGTAGGAACTGCAAC |
| p-SMAD5(m)-R | GAAGCTGAGCAAACTCCTGAT |
| p-SMAD8(m)-F | CGGGTCAGCCTAGCAAGTG |
| p-SMAD8(m)-R | GAGCCGAACGGGAACTCAC |
| Osterix(m)-F | GGAAAGGAGGCACAAAGAAGC |
| Osterix(m)-R | CCCCTTAGGCACTAGGAGC |
| Col1a1(m)-F | GCTCCTCTTAGGGGCCACT |
| Col1a1(m)-R | ATTGGGGACCCTTAGGCCAT |

**Table S3. The main primary antibodies used in this study.**

| **Primary antibodies** | **Company** | **Cat.** | **Dilution** |
| --- | --- | --- | --- |
| BMP6 | Cell Signaling Technology | 70583 | 1:1000 |
| KLF4 | Proteintech | 11880-1-AP | 1:1000 |
| p-SMAD1/5/8 | Cell Signaling Technology | 13820T | 1:1000 |
| SMAD1 | Cell Signaling Technology | 6944 | 1:1000 |
| RUNX2 | Proteintech | 20700-1-AP | 1:1000 |
| MSX2 | Proteintech | 68550-1-Ig | 1:1000 |
| Osterix | Proteintech | 28694-1-AP | 1:1000 |
| α-SMA | Proteintech | 14395-1-AP | 1:2000 |
| CD31 | Proteintech | 11265-1-AP | 1:5000 |
| GAPDH | Proteintech | 10494-1-AP | 1:10000 |
| Col1a1 | Servicebio | GB113041 | 1:1000 |
| TSG101 | Proteintech | 28283-1-AP | 1:5000 |
| CD9 | Proteintech | 20597-1-AP | 1:2000 |
| CD63 | Proteintech | 25682-1-AP | 1:1000 |
| BMPR2 | Proteintech | 14376-1-AP | 1:1000 |
| TGFβ I | Proteintech | 26155-1-AP | 1:1000 |
| TGFβ II | Proteintech | 19999-1-AP | 1:500 |
